# Estimation of induced abortions in Brazil: review of the indirect method calculation based on hospital admissions due to abortion complications

**DOI:** 10.1590/0102-311XEN003025

**Published:** 2025-04-11

**Authors:** Rosa Maria Soares Madeira Domingues, Agatha Sacramento Rodrigues, Marcos Augusto Bastos Dias, Valéria Saraceni, Rejane Sobrinho Pinheiro, Cláudia Medina Coeli, Alberto Pereira Madeiro

**Affiliations:** 1 Instituto Nacional de Infectologia Evandro Chagas, Fundação Oswaldo Cruz, Rio de Janeiro, Brasil.; 2 Universidade Federal do Espírito Santo, Vitória, Brasil.; 3 Instituto Nacional de Saúde da Mulher, da Criança e do Adolescente Fernandes Figueira, Fundação Oswaldo Cruz, Rio de Janeiro, Brasil.; 4 Secretaria Municipal de Saúde, Rio de Janeiro, Brasil.; 5 Instituto de Estudos em Saúde Coletiva, Universidade Federal do Rio de Janeiro, Rio de Janeiro, Brasil.; 6 Centro de Ciências da Saúde, Universidade Estadual do Piauí, Teresina, Brasil.

**Keywords:** Induced Abortion, Hospitalization, Health Information Systems, Aborto Inducido, Hospitalización, Sistemas de Información en Salud

## Abstract

This study aimed to present a new proposal for the calculation of estimates of induced abortion in Brazil using the indirect method, based on hospital admissions due to abortion complications applying the Guttmacher Institute methodology. Data from the Brazilian Hospital Information System of the Brazilian Unified National Health System (SIH/SUS, acronym in Portuguese) were used to identify hospital admissions due to abortion with public funding and from the Brazilian National Agency for Private Health Insurance and Plans (ANS, acronym in Portuguese) for admissions with supplementary health funding. Correction factors were used to exclude hospital admissions due to miscarriage by the woman’s age group, and data from the 2016 National Abortion Survey (PNA, acronym in Portuguese) were used to review the correction factor for complications that did not require hospitalization, based on the simulation of scenarios with different parameters of population coverage of healthcare plans and frequency of induced abortions in SUS and supplementary health patients. Using this new proposal, a total unsafe abortion rate of 8.7 per 1,000 women aged 10-49 was estimated for 2015, with 9.9 in SUS patients and 5,4 in supplementary health patients, and a total unsafe abortion ratio of 18.5 per 100 live births, with 21.1 in SUS patients and 11.2 in supplementary health patients. This new calculation proposal is an advance because it includes data about hospital admissions of supplementary health and a correction factor for complications that did not result in hospitalization using empirical data from the 2016 PNA. This factor is based on available data and is expected to be improved as new evidence is generated, especially evidence that reflects the specificities of the various regions of the country.

## Resumen

Este estudio tuvo como objetivo presentar una nueva propuesta para el cálculo de estimaciones de aborto inducido en Brasil por el método indirecto, a partir de ingresos hospitalarios por complicaciones del aborto, utilizando la metodología del Instituto Guttmacher. Se utilizaron datos del Sistema de Información Hospitalaria del Sistema Único de Salud (SIH/SUS) para identificar las internaciones por aborto con financiamiento público y de la Agencia Nacional de Salud Complementaria (ANS, por su sigla en portugués) para aquellas con financiamiento de la salud suplementaria. Se utilizaron factores de corrección para descontar las hospitalizaciones por abortos espontáneos por grupo de edad de la mujer y datos de la *Encuesta Nacional de Aborto* (PNA, por su sigla en portugués) 2016 para revisar el factor de corrección por complicaciones que no requirieron hospitalización, a partir de la simulación de escenarios con diferencias de parámetros en cobertura poblacional de los planes de salud y frecuencia de abortos inducidos en usuarias del SUS y salud suplementaria. Con esta nueva propuesta, se estimó para el 2015, una tasa total de abortos inducidos de 8,7 por 1.000 mujeres de 10-49 años, y 9,9 de las usuarias del SUS y 5,4 en usuarias de la salud suplementaria, y una ratio total de abortos inducidos de 18,5 por 100 nacidos vivos, con valores de 21,1 en usuarias del SUS y de 11,2 en usuarias del salud suplementaria. Esta nueva propuesta de cálculo avanza incorporando datos de hospitalización del salud suplementaria y un factor de corrección de complicaciones que no resultaron en hospitalización con base en datos empíricos de la PNA del 2016. Este factor se basa en datos actualmente disponibles y se espera que pueda mejorarse a medida que se produzcan nuevas evidencias, especialmente aquella que retrata las particularidades y especificidades de las diferentes regiones del país.

## Introduction

Brazil has restrictive laws on voluntary termination of pregnancy, with induced abortion being legally allowed in only three situations: rape, risk to the mother’s life, and anencephaly ^1^ ^,^ ^2^. However, legal restrictions have not prevented abortion. Data from the *National Abortion Surveys* (PNA, acronym in Portuguese) conducted in 2010 ^3^ and 2016 ^4^ estimate that one in five Brazilian women has had an abortion by the age of 40. The 2016 PNA estimated that 503,000 women had an abortion in 2015 ^4^.

However, clandestine abortion increases the insecurity of abortion. Although the number of hospitalizations due to abortion complications in Brazil decreased from 1995 to 2018 ^5^ ^,^ ^6^ ^,^ ^7^, studies still indicate a high number of moderate and severe complications in hospitalizations due to abortion ^8^, and a higher incidence of severe complications in hospitalizations due to abortion than in hospitalizations due to childbirth ^9^ ^,^ ^10^. Abortion continues to be the fourth leading cause of maternal mortality from direct obstetric causes in the country ^11^ and is more common among young, black, single women with low levels of education ^12^ − and this number is probably underestimated ^13^ ^,^ ^14^.

The study on abortion, including an estimation of its incidence, presents several methodological challenges, which are aggravated by the stigma associated with this theme ^15^. One of the methods used to estimate the number of induced abortions is the indirect estimation. This estimation is based on the number of hospitalizations due to abortion complications, applying correction factors for hospitalizations due to miscarriage and complications that did not result in hospitalization. This methodology has already been used in Brazilian studies, the most recent of which conducted for 1995-2013 ^5^ ^,^ ^6^. These studies used data on hospitalizations in public hospitals obtained from the Brazilian Hospital Information System of the Brazilian Unified National Health System (SIH/SUS, acronym in Portuguese). Data on hospitalizations in private hospitals were not available, so these hospitalizations were estimated using correction factors based on health insurance coverage in the country.

This study aimed to present a new proposal for the calculation of estimates of induced abortions by the indirect method for Brazil, based on a review of correction factors used previously.

## Methods

This is a cross-sectional study that used unidentified and freely accessible databases of SIH/SUS and hospital admissions for supplementary health provided by the Brazilian National Regulatory Agency for Private Health Insurance and Plans (ANS, acronym in Portuguese), both for 2015; the number of patients of health insurance provided by the ANS for 2015; and estimates from the 2016 PNA ^4^ for the number of women who had an abortion in 2015, as a proxy variable for the number of abortions.

### Calculation by indirect method using data on hospitalizations due to abortion complications

In this method, proposed by the Guttmacher Institute (United States), the number of induced abortions is estimated by identifying hospitalizations due to abortion complications (after excluding hospitalizations due to ectopic pregnancy, molar pregnancy, and abnormal products of conception), with the application of two correction factors: one to exclude complications due to miscarriages (spontaneous abortions) and one for abortion complications that did not result in hospitalization. Then the total number of induced abortions is divided by the female population aged 10-49 and multiplied by 1,000 to obtain the induced abortion rate per 1,000 women of reproductive age, or divided by the number of live births and multiplied by 100 to obtain the induced abortion ratio per 100 live births ^16^. The induced abortion rate represents the level of induced abortions in a population of women of reproductive age, while the induced abotion ratio represents the probability of a pregnancy to end in an induced abortion rather than a live birth ^17^.

This study consisted of six steps, which were: (1) access data on the number of hospitalizations due to abortion with public funding; (2) estimate the number of hospitalizations due to abortion in supplementary health; (3) estimate the correction factor to exclude hospitalizations due to miscarriages; (4) estimate the correction factor for complications that did not result in hospitalization; (5) estimate the number of induced abortions; (6) calculate the induced abortion rate per 1,000 women of reproductive age and the induced abortion ratio per 100 live birth.

#### Step 1 - access data on the number of hospitalizations due to abortion with public funding

Unidentified and publicly available data from the SIH/SUS were collected using the microdatasus (https://github.com/rfsaldanha/microdatasus) package of R (http://www.r-project.org) ^18^. The SIH/SUS was implemented in 1990 and it is mainly focused on the payment of hospitalizations in public hospitals and private hospitals affiliated with the SUS.

Hospital admissions due to abortion complications (International Classification of Diseases, 10th revision/ICD-10 O03 to O08) were identified, provided in any AIH field that contained a diagnosis record (principal diagnosis, secondary diagnoses, ICD_death, ICD_associated, ICD_notification) among women aged 10-49 years, according to the municipality of residence and that occurred in 2015. ICD-10 O00, O01, and O02, related to ectopic pregnancy, molar pregnancy, and abnormal products of conception, were not used because they are not probably related to hospital admissions due to induced abortion, a criterion already adopted in previous studies ^19^ ^,^ ^20^. Table 1 shows the hospitalizations due to abortion identified in the SIH/SUS, by age group, and Federative Unit (UF, acronym in Portuguese) of Brazil for 2015.

**Table 1:** Hospitalizations due to abortion identified in the Brazilian Hospital Information System of Brazilian Unified National Health System (SIH/SUS, acronym in Portuguese) by age group and Federative Unit (UF, acronym in Portuguese). Brazil, 2015.

UF	Age group (years)				Total
	10-19	20-29	30-39	40-49	
Acre	249	510	322	66	1,147
Alagoas	686	1,358	781	167	2,992
Amazonas	616	1,242	747	173	2,778
Amapá	252	677	434	78	1,441
Bahia	2,264	5,528	4,100	772	12,664
Ceará	1,332	3,460	2,405	618	7,815
Distrito Federal	264	726	661	147	1,798
Espírito Santo	374	991	730	179	2,274
Goiás	538	1,384	927	175	3,024
Maranhão	1,123	2,498	1,216	186	5,023
Minas Gerais	2,036	5,699	4,453	1,065	13,253
Mato Grosso do Sul	282	620	417	78	1,397
Mato Grosso	324	774	450	91	1,639
Pará	1,467	3,028	1,396	264	6,155
Paraíba	639	1,520	1,156	250	3,565
Pernambuco	1,612	3,751	2,441	519	8,323
Piauí	467	1,001	618	127	2,213
Paraná	759	1,887	1,384	374	4,404
Rio de Janeiro	2,049	5,160	3,452	699	11,360
Rio Grande do Norte	549	1,269	898	218	2,934
Rondônia	268	737	424	88	1,517
Roraima	173	448	282	50	953
Rio Grande do Sul	663	1,783	1,575	445	4,466
Santa Catarina	454	1,286	1,034	231	3,005
Sergipe	522	1,243	857	169	2,791
São Paulo	3,602	10,122	7,699	2,011	23,434
Tocantins	348	786	460	95	1,689
Brazil	23,912	59,488	41,319	9,335	134,054

#### Step 2 - estimate the number of hospitalizations due to abortion in supplementary health

In previous studies, the number of hospitalizations due to abortion in private hospitals was estimated using a correction factor that considered the level of healthcare insurance coverage in Brazil. In other words, based on the number of hospitalizations in the public sector, the number of hospitalizations due to abortion in the private sector was estimated according to health insurance coverage. Previous national studies used a correction factor of 12.5% for the country, considering that it was the coverage of national healthcare insurance ^5^ ^,^ ^6^. In Rio de Janeiro State, a study that estimated the number of unsafe abortions based on the number of hospitalizations refined the use of this correction factor, using different values by age group, given that health insurance coverage increases with the age of the patient ^20^.

However, since 2015, data on hospitalizations performed in the supplementary health have been available in an unidentified and free form on the ANS website, which enables direct access to the number of hospitalizations due to abortion funded by healthcare insurance. These data are published on a monthly basis by UF, with information on the municipality of residence of the woman. Two databases are provided: HOSP_CONS, a consolidated database that shows the ICD-10 of the hospitalization, among other information; and HOSP_DET, which details the procedures performed. The relationship between the two databases can be established using the variable ID_HEALTH_CARE_EVENT.

However, by court order, health insurance companies are not required to report the ICD-10 of the hospitalizations. At the national level, the rate of unreported ICD-10 is 30%, but it varies considerably by UF, from 15.4% in Paraná State to 97.8% in Roraima State (data not shown in the table). Obstetrics has the lowest rate of unreported ICD-10 (23.8% in 2015), but it also varies considerably by UF (12.2% to 84.3%) (data not shown in the table).

To estimate the underreporting of hospitalizations due to abortion in supplementary health using only the hospitalization ICD-10, we compared the number of hospitalizations identified by the ICD-10 to the number of hospitalizations identified by the ICD-10 and/or procedure related to abortion. In the HOSP_CONS and HOSP_DET databases, hospitalizations of women aged 10-49 years in 2015 were selected ^21^. In HOSP_CONS, women who had ICD-10 O03 to O08 records in any of the four existing ICD-10 fields (ICD_1, ICD_2, ICD_3 OR ICD_4) and did not have ICD-10 O00 to O02 records in the same fields were selected. In HOSP_DET, hospitalizations with the identification of the procedure 31309020 - manual vacuum aspiration (MVA) after abortion “31303013” - MVA or “31309062” - curettage after abortion were selected. The two databases were linked using the variable ID_HEALTH_CARE_EVENT to avoid double counting of women who had an ICD-10 and an abortion procedure. Women who had abortion with ICD-10 O00 to O02 were excluded.

Table 2 compares the number of hospitalizations for abortion using only the hospitalization ICD-10 with the number of hospitalizations considering the hospitalization ICD-10 and/or abortion procedure. There was a national increase of 100% in hospitalizations when incorporating procedures, with variations by UF and age group.

**Table 2:** Comparison between the number of hospitalizations due to abortion in supplementary health care using only the diagnosis of hospitalization due to abortion (International Classification of Disease, 10th revision − ICD-10) or the diagnosis plus procedures related to abortion, by Federative Unit (UF, acronym in Portuguese) and age group. Brazil, 2015.

UF	Hospitalizations due to abortion (ICD-10 only)					Hospitalizations due to abortion (ICD-10 and/or procedure)					Ratio of hospitalization (ICD-10 and/or procedure/hospitalization ICD-10)				
	10-19	20-29	30-39	40-49	Total	10-19	20-29	30-39	40-49	Total	10-19	20-29	30-39	40-49	Total
Acre	0	0	0	0	0	0	10	9	4	23	-	-	-	-	-
Alagoas	0	20	38	8	66	3	56	79	13	151	-	2.8	2.1	1.6	2.3
Amazonas	2	19	24	4	49	6	61	82	18	167	3.0	3.2	3.4	4.5	3.4
Amapá	0	13	7	6	26	0	24	16	10	50	-	1.8	2.3	1.7	1.9
Bahia	16	194	282	66	558	36	361	566	129	1,092	2.3	1.9	2.0	2.0	2.0
Ceará	3	59	79	13	154	10	210	364	70	654	3.3	3.6	4.6	5.4	4.2
Federal District	12	64	126	24	226	16	130	282	71	499	1.3	2.0	2.2	3.0	2.2
Espírito Santo	6	105	200	24	335	11	183	327	38	559	1.8	1.7	1.6	1.6	1.7
Goiás	10	104	150	31	295	19	224	290	52	585	1.9	2.2	1.9	1.7	2.0
Maranhão	6	58	94	31	189	12	94	156	41	303	2.0	1.6	1.7	1.3	1.6
Minas Gerais	49	455	743	134	1,381	68	712	1,291	227	2,298	1.4	1.6	1.7	1.7	1.7
Mato Grosso do Sul	3	56	70	23	152	8	91	121	33	253	2.7	1.6	1.7	1.4	1.7
Mato Grosso	3	16	27	4	50	4	55	80	14	153	1.3	3.4	3.0	3.5	3.1
Pará	6	52	49	13	120	25	160	206	31	422	4.2	3.1	4.2	2.4	3.5
Paraíba	0	8	20	1	29	3	66	102	23	194	-	8.3	5.1	23.0	6.7
Pernambuco	6	41	67	14	128	19	155	289	48	511	3.2	3.8	4.3	3.4	4.0
Piauí	1	18	26	0	45	8	59	91	13	171	8.0	3.3	3.5	-	3.8
Paraná	23	190	291	37	541	45	396	644	100	1,185	2.0	2.1	2.2	2.7	2.2
Rio de Janeiro	32	302	501	101	936	67	621	1.031	221	1,940	2.1	2.1	2.1	2.2	2.1
Rio Grande do Norte	0	11	17	2	30	10	113	221	49	393	-	10.3	13.0	24.5	13.1
Rondônia	1	20	35	5	61	6	74	110	20	210	6.0	3.7	3.1	4.0	3.4
Roraima	0	1	0	0	1	1	7	11	1	20	-	7.0	-	-	20.0
Rio Grande do Sul	7	85	157	23	272	12	161	305	52	530	1.7	1.9	1.9	2.3	1.9
Santa Catarina	2	84	139	22	247	14	261	506	82	863	7.0	3.1	3.6	3.7	3.5
Sergipe	0	4	13	1	18	1	33	66	11	111	-	8.3	5.1	11.0	6.2
São Paulo	124	1301	2,025	423	3,873	263	2.368	3.920	835	7,386	2.1	1.8	1.9	2.0	1.9
Tocantins	2	7	8	1	18	2	11	16	4	33	1.0	1.6	2.0	4.0	1.8
Brazil	314	3287	5,188	1,011	9,800	669	6,696	11,181	2,210	20,756	2.1	2.0	2.2	2.2	2.1

Source: Brazilian National Regulatory Agency for Private Health Insurance and Plans ^21^.

To propose a better estimate of hospitalizations due to abortion in supplementary health, the number of hospitalizations due to abortion (hospitalizations with ICD-10 for abortion and/or with specific abortion procedures) was compared to the estimates obtained using the correction factor, which was calculated using the health insurance coverage of patients by UF. To calculate the coverage, the number of women aged 10-49 years with health insurance in 2015 was considered by UF. The number of patients was obtained from the ANS website ^22^ and the female population aged 10-49 from the Brazilian Health Informatics Department (DATASUS) ^23^.

Table 3 shows the population estimates, the median number of patients with health insurance for the four months available (March, June, September, and December 2015) and the estimated coverage of health insurance by age group and UF. The national population coverage of health insurance was 25.88%, with significant variation by UF and age group, with higher coverage in the South and Southeast regions and lower coverage among women aged under 20 years.

**Table 3:** Health insurance coverage by age group and Federative Unit (UF). Brazil, 2015.

UF	10-19 years			20-29 years			30-39 years			40-49 years			Total coverage (%)
	Female population	Beneficiaries *	Coverage ** (%)	Female population	Beneficiaries *	Coverage ** (%)	Female population	Beneficiaries *	Coverage ** (%)	Female population	Beneficiaries *	Coverage ** (%)	
Acre	87,950	2,904	3.30	74,090	3,882	5.24	64,283	4,453	6.93	43.364	3,484	8.03	5.46
Alagoas	296,841	25,769	8.68	281,722	39,616	14.06	260,601	43,052	16.52	206.263	30,266	14.67	13.27
Amazonas	389,492	39,328	10.10	349,541	54,025	15.46	304,570	60,736	19.94	206.628	35,634	17.25	15.18
Amapá	78,890	5,713	7.24	70,459	5,991	8,50	59.721	7,515	12.58	40.342	5,834	14.46	10.04
Bahia	1,219,911	91,460	7.50	1,241,726	141,584	11.40	1,263,222	197,133	15.61	953.630	130,797	13.72	11.99
Ceará	758,539	76,010	10.02	802,076	136,466	17.01	714,170	138,453	19.39	575.476	87,654	15.23	15.39
Federal District	223,702	51,706	23.11	248,727	86,307	34.70	271,447	112,325	41.38	206.898	74,597	36.05	34.18
Espírito Santo	297,576	58,356	19.61	317,985	111,641	35.11	327,428	124,044	37.88	262.730	75,579	28.77	30.66
Goiás	535,435	65,694	12.27	573,878	122,140	21.28	571,679	127,146	22.24	468.231	78,574	16.78	18.31
Maranhão	664,428	28,570	4.30	614,596	47,539	7.73	538,950	52,863	9.81	375.789	32,464	8.64	7.36
Minas Gerais	1,581,488	316,290	20.00	1,668,846	476,535	28.55	1,662,384	558,375	33.59	1.418.977	388,739	27.40	27.48
Mato Grosso do Sul	218,096	44,970	20.62	222,737	55,410	24.88	214,047	70,861	33.11	176.703	52,702	29.83	26.93
Mato Grosso	274,073	32,509	11.86	292,514	57,467	19.65	279,883	61,185	21.86	217.822	38,713	17.77	17.84
Pará	802,628	60,139	7.49	739,917	83,848	11.33	648,529	92,093	14.20	454.149	58,839	12.96	11.15
Paraíba	327,903	24,065	7.34	332,233	39,206	11.80	321,036	43,201	13.46	261.416	28,509	10.91	10.86
Pernambuco	783,945	84,099	10.73	790,061	136,850	17.32	771,854	157,463	20.40	627.699	105,321	16.78	16.27
Piauí	284,770	174,14	6.12	281,342	30,546	10.86	265,150	32,152	12.13	203.749	19,728	9.68	9.65
Paraná	862,708	172,845	20.04	905,295	265,512	29.33	884,168	305,329	34.53	797.258	219,914	27.58	27.94
Rio de Janeiro	1,244,804	342,320	27.50	1,357,429	464,077	34.19	1,407,437	599,840	42.62	1.236.127	452,600	36.61	35.43
Rio Grande do Norte	272,817	31,412	11.51	291,627	52,421	17.98	274,459	59,407	21.65	223.083	38,316	17.18	17.10
Rondônia	148,553	102,88	6.93	148,515	17,726	11.94	138,996	18,097	13,02	107.401	13,500	12.57	10.97
Roraima	56,401	2,372	4.21	48,275	2,923	6.05	41,742	3,389	8,12	28.064	2,360	8.41	6.33
Rio Grande do Sul	802,260	145,447	18.13	857,328	245,741	28.66	857,635	297,522	34,69	765.723	204,305	26.68	27.20
Santa Catarina	501,489	80,636	16.08	595,609	150,306	25.24	570,659	175,366	30,73	485.263	114,826	23.66	24.20
Sergipe	191,466	18,852	9.85	197,089	29,287	14.86	186,700	34,906	18,70	147.418	22,229	15.08	14.57
São Paulo	3.245.524	1,086,360	33.47	3,562,134	1,621,381	45.52	3,806,391	1,998,065	52,49	3.184.132	1,410,407	44.29	44.33
Tocantins	13,2791	6,231	4.69	129,782	10,620	8.18	119,059	119,72	10,06	88.405	7,202	8.15	7.66
Brazil	16,284,480	2,921,759	17.94	16,995,533	4,489,047	26.41	16,826,200	5,386,943	32,02	13.762.740	3,733,093	27.12	25.88

Source: beneficiaries of health insurance ^22^; female population ^23^.

* Beneficiaries of health insurance;

** Coverage of health insurance.

Table 4 shows the comparison of the estimated number of abortions in the supplementary health using the correction factor for health insurance coverage by UF to data obtained from the hospitalization databases provided by the ANS. To estimate abortions using the correction factor, we multiplied the number of hospitalizations due to abortion (ICD-10 O03 to O08) in the SIH/SUS by the health insurance coverage by age group in each UF.

**Table 4:** Number of hospitalizations due to abortion by age group and Federative Unit (UF, acronym in Portuguese), using records from the Brazilian National Regulatory Agency for Private Health Insurance and Plans (ANS, acronym in Portuguese) database or estimates using a correction factor. Brazil, 2015.

UF	Hospitalizations due to abortion (ICD-10 and/or procedure) from supplementary health care database					Estimated number of hospitalizations due to abortion in supplementary health using a correction factor *					Ratio of estimated abortions/reported abortions				
	10-19 years	20-29 years	30-39 years	40-49 years	Total	10-19 years	20-29 years	30-39 years	40-49 years	Total	10-19 years	20-29 years	30-39 years	40-49 years	Total
Acre	0	10	9	4	23	8	27	22	5	63	-	2.67	2.48	1.33	2.72
Alagoas	3	56	79	13	151	60	191	129	25	404	19.85	3.41	1.63	1.88	2.68
Amazonas	6	61	82	18	167	62	192	149	30	433	10.37	3.15	1.82	1.66	2.59
Amapá	0	24	16	10	50	18	58	55	11	142	-	2.40	3.41	1.13	2.83
Bahia	36	361	566	129	1.092	170	630	640	106	1.546	4.71	1.75	1.13	0.82	1.42
Ceará	10	210	364	70	654	133	589	466	94	1.283	13.35	2.80	1.28	1.34	1.96
Federal District	16	130	282	71	499	61	252	274	53	639	3.81	1.94	0.97	0.75	1.28
Espírito Santo	11	183	327	38	559	73	348	277	51	749	6.67	1.90	0.85	1.36	1.34
Goiás	19	224	290	52	585	66	295	206	29	596	3.47	1.32	0.71	0.56	1.02
Maranhão	12	94	156	41	303	48	193	119	16	377	4.02	2.06	0.76	0.39	1.24
Minas Gerais	68	712	1.291	227	2.298	407	1.627	1.496	292	3.822	5.99	2.29	1.16	1.29	1.66
Mato Grosso do Sul	8	91	121	33	253	58	154	138	23	374	7.27	1.69	1.14	0.70	1.48
Mato Grosso	4	55	80	14	153	38	152	98	16	305	9.61	2.76	1.23	1.16	1.99
Pará	25	160	206	31	422	110	343	198	34	685	4.40	2.14	0.96	1.10	1.62
Paraíba	3	66	102	23	194	47	179	156	27	409	15.63	2.72	1.53	1.19	2.11
Pernambuco	19	155	289	48	511	173	650	498	87	1.408	9.10	4.19	1.72	1.81	2.75
Piauí	8	59	91	13	171	29	109	75	12	224	3.57	1.84	0.82	0.95	1.31
Paraná	45	396	644	100	1.185	152	553	478	103	1.287	3.38	1.40	0.74	1.03	1.09
Rio de Janeiro	67	621	1.031	221	1.940	563	1.764	1.471	256	4.055	8.41	2.84	1.43	1.16	2.09
Rio Grande do Norte	10	113	221	49	393	63	228	194	37	523	6.32	2.02	0.88	0.76	1.33
Rondônia	6	74	110	20	210	19	88	55	11	173	3.09	1.19	0.50	0.55	0.82
Roraima	1	7	11	1	20	7	27	23	4	62	7.28	3.88	2.08	4.20	3.08
Rio Grande do Sul	12	161	305	52	530	120	511	546	119	1.296	10.02	3.17	1.79	2.28	2.45
Santa Catarina	14	261	506	82	863	73	325	318	55	770	5.21	1.24	0.63	0.67	0.89
Sergipe	1	33	66	11	111	51	185	160	25	422	51.40	5.60	2.43	2.32	3.80
São Paulo	263	2,368	3,920	835	7,386	1,206	4,607	4,041	891	10,745	4.58	1.95	1.03	1.07	1.45
Tocantins	2	11	16	4	33	16	64	46	8	135	8.16	5.85	2.89	1.93	4.08
Brazil	669	6,696	11,181	2,210	20,756	4,290	15,713	13,228	2,532	35,763	6.41	2.35	1.18	1.15	1.72

ICD-10: International Classification of Diseases, 10th revision.

* Estimate obtained by multiplying the number of hospitalizations due to abortion from the Brazilian Hospital Information System of Brazilian Unified National Health System (SIH/SUS, acronym in Portuguese) (Table 1) by the coverage of health insurance (Table 3) by UF and age group.

The use of the correction factor overestimated the number of hospitalizations due to abortion in the supplementary health at the national level, with an estimated number 1,72 times higher than the reported number. Higher estimates were observed mainly in the younger age groups, in which the estimated number of hospitalizations was 3 to 51 times higher than the reported number among women aged under 20 years. Several states presented a value higher than the national average, with Tocantins (4.08), Sergipe (3.80) and Roraima (3.08) presenting the highest values, while two states presented an estimated number of hospitalizations slightly lower than the reported number (Rondônia 0.82 and Santa Catarina 0.89). Considering the values obtained, we chose to use the reported number of hospitalizations due to abortion in the supplementary health, identified by the ICD-10 of hospitalization and/or procedure related to abortion.

#### Step 3 - estimate the correction factor to exclude hospitalizations due to miscarriages

To exclude hospitalizations due to miscarriages complications, different correction factors were used according to the age group of the woman, because a higher probability of miscarriage has been observed in women aged over 30 years in prospective studies ^24^ ^,^ ^25^: correction factor 0.90 for women < 30 years of age; 0.85 for women aged 30-39 years; and 0.75 for women aged 40 years and older.

These correction factors were applied separately to hospitalizations due to abortion in the public and private sectors, because the distribution by age group was different in the two sectors, with a higher number of hospitalizations among younger women in the public sector and among women aged over 30 years in the private sector (Tables 1 and 2).

#### Step 4 - estimate the correction factor for complications that did not result in hospitalization

To correct abortions that did not result in hospital admission, the method proposed by the Guttmacher Institute recommends using expert opinion to estimate the type of provider and method used, the complication rate for each method, and the number of hospitalizations in case of complications for four groups: urban poor, urban non-poor, rural poor, and rural non-poor populations ^16^.

We used data from the 2016 PNA to guide the selection of the correction factor, with subsequent consultation with experts to discuss the values obtained. The PNA used a direct method to estimate the number of abortions, through interviews with women and the ballot box method, a data collection strategy designed to reduce underreporting of induced abortion, as it is done in an unidentified manner, preserving the anonymity of the woman ^4^. The 2016 PNA estimate of the number of women who had an abortion in 2015 was therefore used as a reference value to define the correction factor, based on its comparison to the number of hospitalizations due to abortion in the public and private sectors.

Some scenarios were simulated to obtain correction factors that would include variations in the parameters used. Table 5 shows the comparison of these scenarios. In all scenarios, we considered only hospitalizations of women aged 18-39 years, the age group adopted in the PNA, and excluded probable hospitalizations due to miscarriage, after applying the specific correction factor for each age group presented in step 3. The 18-19 age group is not available in the ANS database, only the 15-19 age group, so it was necessary to estimate the number of hospitalizations that occur among women aged 18-19 years. According to the PNA, the female population aged 18-39 years in 2015 was 37,287,746. When subtracting women aged 20-39 years (34,032,349) from this number, the remaining 3,255,397 women represent 38% of the estimated population in the 15-19 age group (8,538,652). Since the frequency of abortion in the last year increases with age in the 15-19 age group (according to data from the PNA, 7.6% among girls aged 12-15 years, 10.4% among girls aged 16-17 years, and 11.2% in young women aged 18 and 19) ^4^, we assumed that the number of hospitalizations among women aged 18 and 19 years would be higher than the population distribution, using the parameter of 75% of hospitalizations in the 15-19 age group as an estimate of the number of hospitalizations among women aged 18 and 19 years.

**Table 5:** Correction factor for abortion complications that did not result in hospitalization in the Brazilian Unified National Health System (SUS, acronym in Portuguese) and in the supplementary health system, in women aged 18-39 years. Brazil, 2015.

	Total number of women who reported abortion in the last year estimated by the 2016 PNA	Estimated number of women who reported abortions in the PNA among SUS patients *	Estimated number of women who reported abortions in the PNA among supplementary health patient *	Number of hospitalizations due to abortion in the SUS after application of the miscarriage correction factor **	Number of hospitalizations due to abortion in the supplementary health after application of the miscarriage correction factor **	Total number of hospitalizations due to abortion after application of the miscarriage correction factor **	Correction factor for SUS	Correction factor for supplementary health	Total correction factor
	(a)	(b)	(c)	(d)	(e)	(f)	(b/d)	(c/e)	(a/f)
Scenario 1	503,000	-	-	103,623	15,963	119,586	-	-	4.2
Scenario 2		371,208	128,803	103,623	15,963	-	3.6	8.1	-
Scenario 3a		428,635	74,365	103,623	15,963	-	4.1	4.7	-
Scenario 3b		399,131	103,869	103,623	15,963	-	3.9	6.5	-
Scenario 3c		383,301	119,699	103,623	15,963	-	3.7	7.5	-

PNA: *National Abortion Survey*.

* To estimate the number of abortions among SUS and supplementary health patients, based on the estimated number of women who had an abortion in the last year obtained by the PNA, the following scenarios were considered: scenario 1: considering the total number of women who had abortions according to the PNA in 2015 and the sum of the total number of hospitalizations due to abortion in the public and private sectors in the same year, after applying the correction factor to exclude hospitalizations due to miscarriage; Scenario 2: health insurance coverage by age group (18% in women aged 18-19 years; 26% in women aged 20-29 years, 32% in women aged 30-39 years) was used to estimate the number of supplementary health and SUS patients, considering the same frequency of women who reported abortion in the last year estimated by the 2016 PNA (1.35%) in both sectors (SUS and supplementary health); scenario 3: health insurance coverage by age group (18% in women aged 18-19 years; 26% in women aged 20-29 years, 32% in women aged 30-39 years) was used to estimate the number of supplementary health and SUS patients, and different frequencies of women who reported abortion in the last year were considered for SUS and supplementary health patients (scenario 3a: frequency of women who reported abortion in the last year in supplementary health = 50% of the frequency in SUS patients [1.56% SUS; 0.78% supplementary health]; scenario 3b: frequency of women who reported abortion in the last year in supplementary health = 75% of the frequency in SUS patients [1.45% SUS, 1.09% supplementary health]; scenario 3c: frequency of women who reported abortion in the last year in supplementary health = 90% of the frequency in SUS patients [1.39% SUS, 1.25% supplementary health];

** To estimate hospitalizations in women aged 18-19 years, it was considered that 75% of hospitalizations in women aged 15-19 years occurred in women aged 18-19 years. Correction factor used to exclude hospitalizations due to miscarriage: 18-29 years old = 0.90; 30 to 39 years old = 0.85 ^24^ ^,^ ^25^.

In scenario 1, we estimated the correction factor by analyzing all women together, that is, without distinguishing between SUS and supplementary health care patients and considering that the abortion profile is similar in these two groups. The total number of women who had abortion in 2015 (503,000) as estimated by the PNA was compared to the total number of public and private hospitalizations due to non-spontaneous abortion among women aged 18-39 years.

In scenario 2, we used health insurance coverage by age group in the population aged 18-39 years to estimate the number of abortions among women treated in the SUS and the supplementary health, considering the same abortion profile in both groups. The PNA estimated an abortion rate of 1.35% in the last year, and we used this value to estimate the number of abortions among SUS and supplementary health patients, with subsequent comparison with the number of hospitalizations due to abortion in each sector, calculating a specific correction factor for SUS and supplementary health patients.

Finally, in scenario 3, we considered that the frequency of abortion is different between patients of the public and private sectors, since the PNA showed that women with higher income and education − a profile also observed among healthcare insurance beneficiaries − have a lower frequency of abortion. We used three different parameters: a 50% lower frequency of abortion among supplementary health care patients (difference observed in the highest and lowest income groups in the PNA), and a 25% and 10% lower frequency, considering that patients of the public and private sectors are not only found in these extremes (Table 5: scenarios 3a, 3b, and 3c, respectively). With these parameters, we estimated the number of women treated by SUS and supplementary health, among the women who reported abortion in the last year estimated by the PNA, and compared it with the hospitalizations reported in the public and private sectors, generating a specific correction factor for each sector.

The different scenarios show a correction factor close to 4 for SUS patients. For supplementary health patients, the correction factor ranges from 4.2 (using a single factor for the total number of SUS and supplementary health hospitalizations) to 8.1, a scenario in which the frequency of abortion is assumed to be similar for SUS and supplementary health patients.

#### Step 5 - estimate the number of induced abortions

The equation to calculate the number of induced abortions is ^16^:

Number of induced abortions = (number of hospitalizations with public funding × correction factor for miscarriages × correction factor for complications without hospitalization) + (number of hospitalizations in the supplementary health × correction factor for miscarriages × correction factor for complications without hospitalization).

The correction factor used to exclude miscarriages varied according to the woman’s age group, as reported in step 3 above. The correction factor for complications that did not result in hospitalization to be used for all age groups was 4 for SUS patients and 5 for supplementary health patients (Table 6). The selection of the correction factor for supplementary health patients considered the most conservative scenario, in which the frequency of abortion among supplementary health patients is 50% lower than among SUS patients.

**Table 6:** Estimation of the number of induced abortions, of the induced abortion rate per 1,000 women aged 10-49 years, and the induced abortion ratio per 100 live births according to the health care system. Brazil, 2015.

Health care system/Woman’s age (years)	Number of hospitalizations due to abortion *	Number of hospitalizations after application of the correction factor for miscarriages **	Correction factor for complications (LL/UL) ***	Estimated number of induced abortions (LL/UL) ^#^	Female population 10-49 years ^##^	Live births of females aged 10-49 years ^##^	Induced abortion rate/1,000 women 10-49 years (LL/UL) ^#^	Induced abortion ratio/100 live birth (LL/UL) ^#^
(a)	(b)	(c)	(d) = b × c	(e)	(f)	(d/e) × 1,000	(d/f) × 100
SUS								
10-29	83,400	75,060						
30-39	41,319	35,121						
40-49	9,335	7,001						
Total	134,054	117,182	4 (3/5)	468,728 (351,546/585,910)	47,418,584	2,221,005	9.9 (7.4/12.4)	21.1 (15.8/26.4)
Supplementary health								
10-29	7,365	6,629						
30-39	11,181	9,504						
40-49	2,210	1,658						
Total	20,756	17,791	5 (4/6)	88,955 (71,164/106,746)	16,450,369	796,198	5.4 (4.3/6.5)	11.2 (8.9/13.4)
Brazil				557,683 (422,710/692,656)	63,868,953	3,017,203	8.7 (6.6/10.8)	18.5 (14.0/23.0)

ANS: Brazilian National Regulatory Agency for Private Health Insurance and Plan; ICD-10: International Classification of Diseases, 10th revision; LL: lower limit; UL: Upper limit; SUS: Brazilian Unified National Health System.

* SUS: number of hospitalizations for abortion corresponds to hospitalizations in the Brazilian Hospital Information System of SUS (SIH/SUS, acronym in Portuguese) with ICD-10 O03 to O08; in the supplementary health: number of hospitalizations for abortion corresponds to hospitalizations in the ANS database with ICD-10 O03 to O08 or procedures “31309020” - manual vaccum aspiration (MVA) after abortion or “31303013” − MVA or “31309062” - curettage after abortion;

** Correction factor differentiated according to age group (10-29 years = 0.90; 30-39 years = 0.85; 40-49 years = 0.75), considering the references used ^24^ ^,^ ^25^;

*** Correction factor shown in Table 5. For supplementary health, considering the more conservative scenario, which takes into account a 50% lower frequency of abortion in supplementary health women compared to the SUS;

^#^ To calculate the lower and upper limits of the estimated total number of induced abortions, the correction factors (LL/UL) presented in parentheses in column c were used. To calculate the lower and upper limits of the induced abortion rate and induced abortion ratio, the estimated total number of induced abortions obtained after applying the LL and UL correction factors was used;

^##^ To estimate the female population and the number of live births users of SS, we used medical health insurance coverage, by age group, in women aged 10-49 years in 2015 (10-19 years = 18,0%; 20-29 years = 26%; 30-39 years = 32%; 40-49 years = 27.0%) available at the ANS website ^22^. Female population and SUS live births were obtained by subtracting health plan beneficiaries from the total number of female population and the total number of live births in 2015. Female population aged 10-49 years was retrieved from Brazilian Health Informatics Department ^23^ and number of live births from Brazilian Health Informatics Department ^33^.

#### Step 6 - calculate the induced abortion rate per 1,000 women of reproductive age and the induced abortion ratio per 100 live birth

The numerator of both indicators was the number of induced abortions estimated in step 5. The denominator of the first indicator used the estimated number of women aged 10-49 years from the 2022 Demographic Census obtained from the DATASUS website ^23^. For the second indicator, the number of live births obtained from the Brazilian Information System on Live Births (SINASC, acronym in Portuguese) was used, extracted by the microdatasus package of R ^18^. To calculate the female population aged 10-49 years and live births assisted by the supplementary health, the healthcare insurance plan by age group obtained from the ANS website was used. For both indicators, the upper limit (UL) and lower limit (LL) were calculated, increasing and decreasing by one unit the correction factor for complications that did not result in hospitalization.

The total induce abortion rate was 8.7 per 1,000 women of reproductive age (LL = 6.6; UL = 10.8), with 9.9 (LL = 7.4; UL = 12.4) for SUS patients and 5.4 (LL = 4.3; UL = 6.5) for supplementary health patients. The total induce abortion ratio was 18.5 per 100 live birth (LL = 14.0; UL = 23.0), with 21.1 (LL = 15.8; UL = 26.4) for SUS patients and 11.2 (LL = 8.9; UL = 13.4) for supplementary health patients.

## Discussion

Abortion is a major public health problem, and quantifying and monitoring its occurrence is a relevant strategy for the formulation and implementation of reproductive health actions and services and for the provision of care to women undergoing abortion. The advantage of the indirect method is that it enables the estimation of the number of induced abortions based on administrative data that are regularly available and free of charge. However, its limitations are related to the quality of records in these databases and the parameters used to exclude hospitalizations due to miscarriages and estimate abortion complications that did not require hospitalization, which will be discussed below.

Hospitalization data in the public sector have been used since the 1990s. A nationwide validation study conducted in 2021-2022 found good coverage of obstetric hospitalization data in the SIH/SUS, with no evidence of underreporting of hospitalizations due to abortion when using the ICD-10 for hospitalization ^26^. Information about hospitalizations in the supplementary health was not available when the most recent national studies were conducted which estimated the number of induced abortions using the indirect method ^5^ ^,^ ^6^. In both studies, a correction factor was used to estimate the number of hospitalizations due to abortion in the supplementary health based on the number of hospitalizations reported in the public sector and the health insurance coverage in the country.

However, using this correction factor involves two problems. First, the use of a single national correction factor disregards the existence of regional inequalities in access to health insurance. Higher coverage rates are observed in the South, Southeast, and Central-West regions ^27^, which can bias estimates, with overreporting of induced abortions in UF with health insurance coverage lower than the national average and underreporting in UF with coverage above the average. Second, the use of this correction factor is based on the idea that the incidence of induced abortions is the same for SUS and supplementary health patients. Data from the 2010 ^3^ and 2016 ^4^ PNAs show that the incidence of abortions is much lower among women with higher levels of education and income, a profile found in supplementary health patients, which makes us believe that the frequency of abortions would be lower among these patients due to greater access to reproductive planning services and lower incidence of unintended pregnancy.

In this study, we used for the first time data on hospital admissions in private services provided by the ANS starting in 2015. Due to the underreporting of the ICD-10 in this database, we also evaluated data on procedures related to abortion, which resulted in a 100% increase in hospital admissions due to abortion in the supplementary health. These records include both inpatient and day-clinic procedures. We emphasize that the use of abortion procedures is not recommended by the Guttmacher Institute methodology ^16^, but was used to correct the underreporting of the ICD-10 identified. However, there may be some overreporting if women admitted for diagnoses related to ICD-10 O00 to O02 underwent abortion procedures and did not have their ICD-10 registered, thus being inappropriately included in the total number of hospitalizations due to abortion.

The comparison of the number of records in these databases with the estimated number of hospitalizations in the supplementary health, calculated using the number of hospitalizations in the public sector and health insurance coverage, showed that the calculation using the correction factor overestimated the number of hospitalizations in the private sector in all UF, except Santa Catarina and Rondônia states. It must be highlighted that, unlike previous studies ^5^ ^,^ ^6^, specific correction factors were used for each UF and age group, which enabled the calculation of more accurate estimates considering the context of each location. 

Also unlike previous national studies ^5^ ^,^ ^6^, we used a specific correction factor by age group to exclude miscarriages. Factors differentiated by age group had already been used in a study conducted in Rio de Janeiro State ^20^, but with different values from those adopted in this study. Since the ANS database only includes the age group of 30-39 years, it was not possible to use specific factors for women over 35 years of age, when the incidence of miscarriage increases. Therefore, miscarriages is probably overestimated in the age group of 30-34 years due to the use of the same correction factor used for women aged 35-39 years.

Finally, the most complex parameter was the correction factor for complications that do not result in hospitalization. Results from the 2010 ^3^ and 2016 ^4^ PNAs indicate that about half of Brazilian women who had induced abortions required hospitalization, with a suggested correction factor of 2. However, the PNA only included women aged 18 to 39 years and did not assess women living in rural areas and who were illiterate. Therefore, of the four subpopulations suggested by the Guttmacher methodology ^16^, only the urban non-poor and urban poor subpopulations would be represented, the latter in an incomplete manner due to the non-inclusion of illiterate women. The estimated number of 503,000 women who had abortions in the 2016 PNA ^4^ is an extrapolation, suggesting that women who were not included have the same frequency of abortions and the same pattern of health service use as interviewed women.

In the current study, several simulations were performed, but none of them obtained a correction factor of 2, as suggested by the 2016 PNA ^4^. In all scenarios, the correction factor for the public sector was close to 4, similar to that used in previous national studies ^5^ ^,^ ^6^. For the supplementary health, the correction factor ranged from 4.2 to 8.1, depending on the assumptions about the population covered by health insurance and the frequency of abortions among SUS and supplementary health patients. For the analysis of scenarios, we considered only hospitalizations of women aged 18-39 years, the age group studied in the PNA. Therefore, we estimated that 75% of hospitalizations in the age group of 15-19 years occurred among young women aged 18-19 years. If a lower number was adopted, the correction factor would be even higher because the number of hospitalizations among women aged 18-19 years would be lower.

The selection of correction factors, especially the one used to correct complications that did not result in hospitalization, is a subject of much debate because it depends on the safety of abortion methods and access to health services ^28^ ^,^ ^29^. Unsafe methods are associated with a high number of women requiring hospitalization ^30^ and a low correction factor. The PNAs showed a decrease in the number of women who used medication for abortion (48% in 2010; 39% in 2021), a safe method with a low complication rate, and in the number of women who were hospitalized to complete the abortion (55% in 2010; 43% in 2021) ^3^ ^,^ ^31^.

Due to the uncertainties of the parameters, the Guttmacher Institute methodology recommends calculating the upper and lower limits, increasing and decreasing by one unit the correction factor for complications that did not result in hospitalization to obtain low and high estimates, and the actual incidence of abortion is expected to be within this range ^16^. For hospitalizations in the public sector, we suggest a correction factor of 4, with a range of 3 to 5, while for the private sector, a correction factor of 5, with a range of 4 to 6.

With the calculations used in this study, we estimated a induced abortion rate of 8.7 per 1,000 women of reproductive age in 2015, a value much lower than the average annual coefficient observed in 1996-2012, of 17.0 per 1,000 women of reproductive age ^5^. However, our upper limit (10.8) was close to the lower limit reported in 2013, of 12 per 1,000 women of reproductive age ^6^. For the induced abortion ratio, our estimate of 18.5 per 100 live birth was half the average value estimated for the period 1996-2012. The downward trend in the induced abortion ratio between 1995 and 2013 ^5^ ^,^ ^6^ could be a possible explanation for the lower induced abortion rate value observed in this study, but not for the induced abortion ratio, which remained stable in 1996-2012. Differences in the calculation methods used in this study, such as the use of hospitalization data from the ANS, specific factors by age group to exclude miscarriages, and different correction factors for hospitalizations in the public sector and in the supplementary health are more plausible reasons for the discrepancies found in this study.

The large difference in the correction factor used for complications that did not result in hospitalization in relation to the correction factor proposed by the 2016 PNA ^4^ has several possible explanations. First, the number of hospitalizations may be underestimated due to registration errors or problems with hospital admission authorizations. It is also possible that in hospitalizations of women with later abortion complications, the ICD of the abortion was not recorded, but the ICD of the complication that led to the hospitalization. Deaths due to abortion are not included in this methodology, which may lead to underreporting if the ICD-10 of the abortion is not included in the hospitalization that led to death. The number of miscarriages in the age group of 30-34 years may be overestimated, reducing the estimated number of hospitalizations due to abortion in this age group in both the SUS and the supplementary health. Regarding abortion estimates, the PNA extrapolated its results to the entire female population aged 18-39 years, a value that we compared to the total number of hospital admissions. The frequency of abortion and the profile of service use may be different among illiterate women and women living in rural areas, resulting in a higher correction factor, especially among SUS patients. Also, women may have inaccurately answered the question about hospitalization in the PNA, which focuses on seeking care and receiving care at health facilities, not necessarily hospitalization. Supplementary health patients may have greater access to safer methods, resulting in less need for post-abortion hospital care. The PNA estimated the number of women who had an abortion, and not the number of abortions, so it did not consider that the same woman could have more than one abortion in the same year, thus underestimating the number of abortions. Finally, hospitalizations in private services with out-of-pocket payments are not included in the supplementary health database, which only shows hospitalizations financed by health insurance.

One issue that deserves attention in future studies is the distribution of abortions by age group. In the simulations performed, only hospitalizations of women aged 18-39 years were considered, as this is the age group analyzed in the PNA. However, data from the 2010, 2016, and 2021 PNAs show that, although the highest frequency of abortion is in the age group of 20-29 years, the first abortion is more common in the age group of 15-19 years ^3^ ^,^ ^4^ ^,^ ^31^. National data show a higher incidence of induced abortion among young people with a higher level of education, as a result of an early pregnancy competing with other personal projects ^32^, and the incidence of abortion may differ between adolescents treated by SUS and those treated by supplementary health.

Finally, in a country as large and diverse as Brazil, it is essential that local and regional studies provide evidence to support the use of correction factors that are more appropriate to each context. More specific data by region of the country and by type of health care provider are required to support the definition of the correction factor. Different values will result in different estimates of the number of induced abortions, limiting the comparison of results.

## Conclusion

The methodology for calculating the number of induced abortions by indirect method, based on hospital admissions due to abortion complications, allows the production of rapid estimates using regular and free administrative databases. In this study, we proposed a revision of the methodology previously used, incorporating the database of hospital admissions from the supplementary sector and using correction factors for miscarriages and abortion complications that did not result in hospitalization in the public and private sectors. This new calculation method estimates the number of induced abortions for the total female population, and separately for SUS and supplementary health patients. We understand this methodology can be improved by refining the correction factors and continuously improving the databases. Future investigations should prioritize the study of abortion among adolescents and obtain parameters for the calculation of correction factors for specific populations.
