# Supplementary material for: Estimation of induced abortions in Brazil: review of the indirect method calculation based on hospital admissions due to abortion complications
Source: Cad Saude Publica. 2025 Apr 11;41(4):e00003025. doi: 10.1590/0102-311XEN003025 (PMC11996186; doi:10.1590/0102-311XEN003025)
Supplement: Translation

# Translation: Estimativa de abortos induzidos no Brasil: revisão do cálculo por método indireto baseado em internações hospitalares por complicações do aborto

**DOI:** 10.1590/0102-311XPT003025

**Authors:** Rosa Maria Soares Madeira Domingues, Agatha Sacramento Rodrigues, Marcos Augusto Bastos Dias, Valéria Saraceni, Rejane Sobrinho Pinheiro, Cláudia Medina Coeli, Alberto Pereira Madeiro

**Affiliations:** 1 Instituto Nacional de Infectologia Evandro Chagas, Fundação Oswaldo Cruz, Rio de Janeiro, Brasil.; 2 Universidade Federal do Espírito Santo, Vitória, Brasil.; 3 Instituto Nacional de Saúde da Mulher, da Criança e do Adolescente Fernandes Figueira, Fundação Oswaldo Cruz, Rio de Janeiro, Brasil.; 4 Secretaria Municipal de Saúde, Rio de Janeiro, Brasil.; 5 Instituto de Estudos em Saúde Coletiva, Universidade Federal do Rio de Janeiro, Rio de Janeiro, Brasil.; 6 Centro de Ciências da Saúde, Universidade Estadual do Piauí, Teresina, Brasil.

**Keywords:** Aborto Induzido, Hospitalização, Sistemas de Informação em Saúde

## Abstract

Este estudo teve como objetivo apresentar a nova proposta de cálculo das estimativas de aborto induzido no Brasil por método indireto, baseada nas internações hospitalares por complicações de aborto, utilizando a metodologia do Instituto Guttmacher. Foram utilizados dados do Sistema de Informação Hospitalar do Sistema Único de Saúde (SIH/SUS) para identificações de internações por aborto com financiamento público e da Agência Nacional de Saúde Suplementar (ANS) para aquelas com financiamento pela saúde suplementar. Foram utilizados fatores de correção para descontar internações por abortos espontâneos por faixa etária da mulher e utilizados dados da *Pesquisa Nacional de Aborto* (PNA) de 2016 para revisão do fator de correção de complicações que não necessitaram de internações, a partir da simulação de cenários com parâmetros distintos de cobertura populacional de planos de saúde e frequência de abortos induzidos em usuárias do SUS e da saúde suplementar. Utilizando essa nova proposta, foi estimada, para 2015, uma taxa de aborto induzido total de 8,7 por 1.000 mulheres de 10-49 anos, sendo de 9,9 em usuárias do SUS e de 5,4 em usuárias da saúde suplementar, e uma razão de aborto induzido total de 18,5 por 100 nascidos vivos, com valores de 21,1 em usuárias do SUS e 11,2 em usuárias da saúde suplementar. Essa nova proposta de cálculo avança ao incorporar dados de internações da saúde suplementar e fator de correção para complicações que não resultaram em internação baseado em dados empíricos da PNA 2016. Esse fator se baseia nos dados atualmente disponíveis e espera-se que possa ser aprimorado à medida que novas evidências sejam produzidas, principalmente as que retratam as particularidades e especificidades das diversas regiões do país.

## Introdução

O Brasil apresenta legislação restritiva para a interrupção voluntária da gravidez, sendo o aborto induzido previsto legalmente em apenas três situações: estupro, risco de vida materno e anencefalia ^1^ ^,^ ^2^. Entretanto, a restrição legal não tem evitado sua prática. Dados da *Pesquisa Nacional de Aborto* (PNA) realizadas nos anos 2010 ^3^ e 2016 ^4^ estimam que uma em cada cinco mulheres brasileiras, aos 40 anos, já haviam realizado um aborto. A PNA de 2016 estimou que 503 mil mulheres realizaram abortos em 2015 ^4^.

A clandestinidade, entretanto, aumenta a insegurança do aborto. Embora as internações por complicações do aborto no país tenham apresentado redução no período 1995-2018 ^5^ ^,^ ^6^ ^,^ ^7^, estudos ainda indicam uma elevada proporção de complicações moderadas e graves em internações por aborto ^8^, bem como maior ocorrência de complicações graves em internações por aborto do que em internações para o parto ^9^ ^,^ ^10^. O aborto continua sendo a quarta causa de mortalidade materna por causas obstétricas diretas no país ^11^, sendo mais frequente em mulheres jovens, negras, solteiras e de baixa escolaridade ^12^, estando esse número provavelmente subestimado ^13^ ^,^ ^14^.

O estudo do aborto, incluindo a estimativa de sua frequência, apresenta várias dificuldades metodológicas, que são agravadas pelo estigma relacionado ao tema ^15^. Um dos métodos utilizados para estimar o número de abortos induzidos é a estimativa indireta, a partir do número de internações por complicações do aborto e aplicação de fatores de correção para internações por abortos espontâneos e para complicações que não resultaram em internação, metodologia já utilizada previamente em estudos nacionais, sendo as últimas realizadas para o período 1995-2013 ^5^ ^,^ ^6^. Nesses estudos, foram utilizados os dados de internações em hospitais públicos, obtidos no Sistema de Informação Hospitalar do Sistema Único de Saúde (SIH/SUS). Dados de internações em hospitais privados não estavam disponíveis, sendo essas internações estimadas a partir de fatores de correção baseados na cobertura de planos de saúde no país.

O objetivo deste estudo foi apresentar uma nova proposta de cálculo das estimativas de aborto induzido por método indireto para o país, a partir da revisão dos fatores de correção utilizados previamente.

## Métodos

Trata-se de estudo seccional, que utilizou bases de dados não identificadas e de acesso livre do SIH/SUS e das internações hospitalares na saúde suplementar disponibilizadas pela Agência Nacional de Saúde Suplementar (ANS), ambas referentes ao ano de 2015; do número de beneficiárias de planos de saúde disponibilizadas pela ANS para o ano de 2015; e estimativas da PNA 2016 ^4^ do número de mulheres que realizaram aborto em 2015, como variável *proxy* para o número de abortos.

### O método de cálculo por estimativa indireta utilizando dados de internação por complicações de aborto

Na metodologia proposta pelo Instituto Guttmacher (Estados Unidos), o número de abortos induzidos é estimado a partir da identificação de internações por complicações do aborto (após exclusão de internações por gestações ectópicas, gestações molares e produtos anormais da concepção), sendo aplicados dois fatores de correção: um para exclusão de complicações por abortos espontâneos e outro para complicações de abortamento que não resultaram em internação hospitalar. O número total de abortos induzidos é, então, dividido pela população feminina de 10-49 anos e multiplicado por 1.000, para se obter a taxa de abortos induzidos por 1.000 mulheres em idade fértil; ou dividido pelo número de nascidos vivos e multiplicado por 100, para se obter a razão de abortos induzidos por 100 nascidos vivos ^16^. A taxa de aborto induzidos expressa o nível de abortos induzidos em uma população feminina em idade reprodutiva, enquanto a razão de abortos induzidos representa a probabilidade de uma gravidez terminar em aborto induzido em vez de um nascimento vivo ^17^.

A seguir, serão apresentadas as seis etapas realizadas neste estudo: (1) obtenção do número de internações por aborto com financiamento público; (2) estimativa do número de internações por aborto na saúde suplementar; (3) estimativa do fator de correção para descontar internações por aborto espontâneo; (4) estimativa do fator de correção para complicações que não resultaram em internação hospitalar; (5) estimativa do número de abortos induzidos; (6) cálculo da taxa de aborto induzidos por 1.000 mulheres em idade fértil e da razão de abortos induzidos por 100 nascidos vivos.

#### Etapa 1 - obtenção do número de internações por aborto com financiamento público

Foram utilizados dados não identificados e de acesso público disponíveis no SIH/SUS captados pelo pacote *microdatasus* (https://github.com/rfsaldanha/microdatasus) por meio da linguagem de programação estatística R (http://www.r-project.org) ^18^. O SIH/SUS foi implantado em 1990 e tem por objetivo principal o pagamento das internações realizadas em hospitais públicos e privados conveniados ao SUS.

Foram identificadas as internações hospitalares por complicações do aborto (Classificação Internacional de Doenças, 10ª revisão − CID-10 O03 a O08), registradas em qualquer campo de Autorização de Internação Hospitalar (AIH) que contivesse registro de diagnóstico (diagnóstico principal, diagnósticos secundários, CID_morte, CID_associada, CID_notificação), de mulheres de 10-49 anos, segundo município de residência, ocorridas em 2015. As CID-10 O00, O01 e O02, relativas a gestações ectópicas, gestações molares e produtos anormais da concepção, não foram utilizadas, por ser pouco provável o seu uso no caso de internações por abortos induzidos, critério já adotado em estudos anteriores ^19^ ^,^ ^20^. As internações por aborto identificadas no SIH/SUS, por faixa etária e Unidade Federativa (UF), para o ano 2015, estão na Tabela 1.

**Tabela 1:** Internações por aborto identificadas no Sistema de Informação Hospitalar do Sistema Único de Saúde (SIH/SUS), por faixa etária e Unidade Federativa (UF). Brasil, 2015.

UF	Faixa etária (anos)			Total	
	10-19	20-29	30-39	40-49	
Acre	249	510	322	66	1.147
Alagoas	686	1.358	781	167	2.992
Amazonas	616	1.242	747	173	2.778
Amapá	252	677	434	78	1.441
Bahia	2.264	5.528	4.100	772	12.664
Ceará	1.332	3.460	2.405	618	7.815
Distrito Federal	264	726	661	147	1.798
Espírito Santo	374	991	730	179	2.274
Goiás	538	1.384	927	175	3.024
Maranhão	1.123	2.498	1.216	186	5.023
Minas Gerais	2.036	5.699	4.453	1.065	13.253
Mato Grosso do Sul	282	620	417	78	1.397
Mato Grosso	324	774	450	91	1.639
Pará	1.467	3.028	1.396	264	6.155
Paraíba	639	1.520	1.156	250	3.565
Pernambuco	1.612	3.751	2.441	519	8.323
Piauí	467	1.001	618	127	2.213
Paraná	759	1.887	1.384	374	4.404
Rio de Janeiro	2.049	5.160	3.452	699	11.360
Rio Grande do Norte	549	1.269	898	218	2.934
Rondônia	268	737	424	88	1.517
Roraima	173	448	282	50	953
Rio Grande do Sul	663	1.783	1.575	445	4.466
Santa Catarina	454	1.286	1.034	231	3.005
Sergipe	522	1.243	857	169	2.791
São Paulo	3.602	10.122	7.699	2.011	23.434
Tocantins	348	786	460	95	1.689
Brasil	23.912	59.488	41.319	9.335	134.054

#### Etapa 2 - estimativa do número de internações por aborto na saúde suplementar

Em estudos anteriores, a estimativa do número de internações por aborto em hospitais privados era feita a partir de um fator de correção que considerava a cobertura de planos de saúde no país. Ou seja, a partir do número de internações no setor público, estimava-se que haveria um número de internações por aborto no setor privado, correspondente à cobertura de planos de saúde. Os estudos nacionais prévios utilizaram fator de correção de 12,5% para o país, considerando que essa era a cobertura de planos de saúde nacional ^5^ ^,^ ^6^. No Rio de Janeiro, uma investigação que estimou o número de abortos inseguros a partir do número de internações refinou o uso desse fator de correção, utilizando valores diferenciados por faixa etária, dado que a cobertura de beneficiárias de planos de saúde aumenta com a idade ^20^.

Porém, desde 2015, dados de internações realizadas na saúde suplementar estão disponíveis de forma não identificada e gratuita no site da ANS, o que permite a obtenção direta do número de internações por aborto com financiamento por planos de saúde. Esses dados são disponibilizados em arquivos mensais, por UF, contendo informação sobre o município de residência da mulher. São disponibilizadas duas bases: a base HOSP_CONS, uma base consolidada que contém, entre outras informações, a CID-10 de internação; e a base HOSP_DET, que detalha os procedimentos realizados. O relacionamento das duas bases pode ser feito pela variável ID_EVENTO_ATENCAO_SAUDE.

Entretanto, por decisão judicial, as operadoras de saúde não são obrigadas a informar a CID-10 de internação. Nacionalmente, a proporção de CID-10 não informada é de 30%, mas apresenta grande variação por UF, de 15,4% no Paraná a 97,8% em Roraima (dado não mostrado em tabela). A especialidade obstetrícia é a que apresenta menor proporção de CID-10 não informada (23,8% em 2015), mas também com grande variação por UF (12,2% a 84,3%) (dado não mostrado em tabela).

Para se obter estimativa do sub-registro de internações por aborto na saúde suplementar ao utilizar apenas a CID-10 de internação, comparamos o número de internações identificadas pela CID-10 com o número das identificadas pela CID-10 e/ou procedimento relacionado ao aborto. Nas bases HOSP_CONS e HOSP_DET, referentes ao ano de 2015 ^21^, foram selecionadas as internações de mulheres de 10-49 anos. Na base HOSP_CONS, foram selecionadas as mulheres que apresentassem registro de CID-10 O03 a O08 em algum dos quatro campos existentes para registro da CID-10 (CID_1, CID_2, CID_3 OU CID_4) e não apresentassem registro de CID-10 O00 a O02 nesses mesmos campos. Na base HOSP_DET, foram selecionadas as internações que apresentassem registro do procedimento “31309020” - aspiração manual intrauterina (AMIU) pós-abortamento ou “31303013” − AMIU ou “31309062” - curetagem pós-abortamento. Foi feito o relacionamento entre as duas bases utilizando a variável ID_EVENTO_ATENCAO_SAUDE para evitar contagem duplicada de mulheres que apresentassem CID-10 e procedimento de aborto. Também foram excluídas mulheres que apresentassem procedimento de aborto, mas registro de CID-10 O00 a O02.

A Tabela 2 apresenta a comparação do número de internações por aborto utilizando apenas a CID-10 de internação com o número de internações considerando CID-10 de internação e/ou procedimento de aborto. Verificou-se aumento nacional de 100% das internações ao incorporar os procedimentos, com variações por UF e faixa etária.

**Tabela 2:** Comparação entre o número de internações por aborto na saúde suplementar utilizando apenas o diagnóstico da internação por aborto (Classificação Internacional de Doenças, 10ª revisão - CID-10) ou o diagnóstico mais procedimentos relacionados ao aborto, por Unidade Federativa (UF) e faixa etária. Brasil, 2015.

UF	Internações por aborto (apenas CID-10)					Internações por aborto (CID-10 e/ou procedimento)					Razão de internação (CID-10 e/ou procedimento/internação CID)				
	10-19 anos	20-29 anos	30-39 anos	40-49 anos	Total	10-19 anos	20-29 anos	30-39 anos	40-49 anos	Total	10-19 anos	20-29 anos	30-39 anos	40-49 anos	Total
Acre	0	0	0	0	0	0	10	9	4	23	-	-	-	-	-
Alagoas	0	20	38	8	66	3	56	79	13	151	-	2,8	2,1	1,6	2,3
Amazonas	2	19	24	4	49	6	61	82	18	167	3	3,2	3,4	4,5	3,4
Amapá	0	13	7	6	26	0	24	16	10	50	-	1,8	2,3	1,7	1,9
Bahia	16	194	282	66	558	36	361	566	129	1.092	2,3	1,9	2,0	2,0	2,0
Ceará	3	59	79	13	154	10	210	364	70	654	3,3	3,6	4,6	5,4	4,2
Distrito Federal	12	64	126	24	226	16	130	282	71	499	1,3	2,0	2,2	3,0	2,2
Espírito Santo	6	105	200	24	335	11	183	327	38	559	1,8	1,7	1,6	1,6	1,7
Goiás	10	104	150	31	295	19	224	290	52	585	1,9	2,2	1,9	1,7	2,0
Maranhão	6	58	94	31	189	12	94	156	41	303	2,0	1,6	1,7	1,3	1,6
Minas Gerais	49	455	743	134	1.381	68	712	1.291	227	2.298	1,4	1,6	1,7	1,7	1,7
Mato Grosso do Sul	3	56	70	23	152	8	91	121	33	253	2,7	1,6	1,7	1,4	1,7
Mato Grosso	3	16	27	4	50	4	55	80	14	153	1,3	3,4	3,0	3,5	3,1
Pará	6	52	49	13	120	25	160	206	31	422	4,2	3,1	4,2	2,4	3,5
Paraíba	0	8	20	1	29	3	66	102	23	194	-	8,3	5,1	23,0	6,7
Pernambuco	6	41	67	14	128	19	155	289	48	511	3,2	3,8	4,3	3,4	4,0
Piauí	1	18	26	0	45	8	59	91	13	171	8,0	3,3	3,5	-	3,8
Paraná	23	190	291	37	541	45	396	644	100	1.185	2,0	2,1	2,2	2,7	2,2
Rio de Janeiro	32	302	501	101	936	67	621	1.031	221	1.940	2,1	2,1	2,1	2,2	2,1
Rio Grande do Norte	0	11	17	2	30	10	113	221	49	393	-	10,3	13,0	24,5	13,1
Rondônia	1	20	35	5	61	6	74	110	20	210	6,0	3,7	3,1	4,0	3,4
Roraima	0	1	0	0	1	1	7	11	1	20	-	7,0	-	-	20,0
Rio Grande do Sul	7	85	157	23	272	12	161	305	52	530	1,7	1,9	1,9	2,3	1,9
Santa Catarina	2	84	139	22	247	14	261	506	82	863	7,0	3,1	3,6	3,7	3,5
Sergipe	0	4	13	1	18	1	33	66	11	111	-	8,3	5,1	11,0	6,2
São Paulo	124	1.301	2.025	423	3.873	263	2.368	3.920	835	7.386	2,1	1,8	1,9	2,0	1,9
Tocantins	2	7	8	1	18	2	11	16	4	33	1,0	1,6	2,0	4,0	1,8
Brasil	314	3.287	5.188	1.011	9.800	669	6.696	11.181	2.210	20.756	2,1	2,0	2,2	2,2	2,1

Fonte: Agência Nacional de Saúde Suplementar ^21^.

A fim de propor uma melhor estimativa das internações por aborto na saúde suplementar, realizou-se comparação do número de internações por aborto (internações com CID-10 de aborto e/ou com procedimentos específicos do aborto) com as estimativas obtidas com o uso do fator de correção, calculado a partir da cobertura de beneficiárias de planos de saúde por UF. Para cálculo da cobertura, considerou-se a proporção de mulheres de 10-49 anos com plano de saúde médico em 2015 por UF. O número de beneficiárias foi obtido pelo site da ANS ^22^ e a população feminina de 10-49 anos do Departamento de Informática do SUS (DATASUS) ^23^.

A Tabela 3 apresenta as estimativas populacionais e a mediana do número de beneficiárias de planos de saúde médicos dos quatro meses disponíveis (março, junho, setembro e dezembro de 2015), além da cobertura estimada de planos de saúde por faixa etária e UF. Verificou-se cobertura populacional nacional de beneficiárias de planos de saúde médicos de 25,88%, com grande variação segundo UF e faixa etária, sendo maior nos estados das regiões Sul e Sudeste e menor em mulheres com menos de 20 anos.

**Tabela 3:** Cobertura de planos de saúde médico por faixa etária e Unidade Federativa (UF). Brasil, 2015.

UF	10-19 anos			20-29 anos			30-39 anos			40-49 anos			Cobertura total (%)
	População feminina	Beneficiárias *	Cobertura ** (%)	População feminina	Beneficiárias *	Cobertura ** (%)	População feminina	Beneficiárias *	Cobertura ** (%)	População feminina	Beneficiárias *	Cobertura ** (%)	
Acre	87.950	2.904	3,30	74.090	3.882	5,24	64.283	4.453	6,93	43.364	3.484	8,03	5,46
Alagoas	296.841	25.769	8,68	281.722	39.616	14,06	260.601	43.052	16,52	206.263	30.266	14,67	13,27
Amazonas	389.492	39.328	10,10	349.541	54.025	15,46	304.570	60.736	19,94	206.628	35.634	17,25	15,18
Amapá	78.890	5.713	7,24	70.459	5.991	8,50	59.721	7.515	12,58	40.342	5.834	14,46	10,04
Bahia	1.219.911	91.460	7,50	1.241.726	141.584	11,40	1.263.222	197.133	15,61	953.630	130.797	13,72	11,99
Ceará	758.539	76.010	10,02	802.076	136.466	17,01	714.170	138.453	19,39	575.476	87.654	15,23	15,39
Distrito Federal	223.702	51.706	23,11	248.727	86.307	34,70	271.447	112.325	41,38	206.898	74.597	36,05	34,18
Espírito Santo	297.576	58.356	19,61	317.985	111.641	35,11	327.428	124.044	37,88	262.730	75.579	28,77	30,66
Goiás	535.435	65.694	12,27	573.878	122.140	21,28	571.679	127.146	22,24	468.231	78.574	16,78	18,31
Maranhão	664.428	28.570	4,30	614.596	47.539	7,73	538.950	52.863	9,81	375.789	32.464	8,64	7,36
Minas Gerais	1.581.488	316.290	20,00	1.668.846	476.535	28,55	1.662.384	558.375	33,59	1.418.977	388.739	27,40	27,48
Mato Grosso do Sul	218.096	44.970	20,62	222.737	55.410	24,88	214.047	70.861	33,11	176.703	52.702	29,83	26,93
Mato Grosso	274.073	32.509	11,86	292.514	57.467	19,65	279.883	61.185	21,86	217.822	38.713	17,77	17,84
Pará	802.628	60.139	7,49	739.917	83.848	11,33	648.529	92.093	14,20	454.149	58.839	12,96	11,15
Paraíba	327.903	24.065	7,34	332.233	39.206	11,80	321.036	43.201	13,46	261.416	28.509	10,91	10,86
Pernambuco	783.945	84.099	10,73	790.061	136.850	17,32	771.854	157.463	20,40	627.699	105.321	16,78	16,27
Piauí	284.770	17.414	6,12	281.342	30.546	10,86	265.150	32.152	12,13	203.749	19.728	9,68	9,65
Paraná	862.708	172.845	20,04	905.295	265.512	29,33	884.168	305.329	34,53	797.258	219.914	27,58	27,94
Rio de Janeiro	1.244.804	342.320	27,50	1.357.429	464.077	34,19	1.407.437	599.840	42,62	1.236.127	452.600	36,61	35,43
Rio Grande do Norte	272.817	31.412	11,51	291.627	52.421	17,98	274.459	59.407	21,65	223.083	38.316	17,18	17,10
Rondônia	148.553	10.288	6,93	148.515	17.726	11,94	138.996	18.097	13,02	107.401	13.500	12,57	10,97
Roraima	56.401	2.372	4,21	48.275	2.923	6,05	41.742	3.389	8,12	28.064	2.360	8,41	6,33
Rio Grande do Sul	802.260	145.447	18,13	857.328	245.741	28,66	857.635	297.522	34,69	765.723	204.305	26,68	27,20
Santa Catarina	501.489	80.636	16,08	595.609	150.306	25,24	570.659	175.366	30,73	485.263	114.826	23,66	24,20
Sergipe	191.466	18.852	9,85	197.089	29.287	14,86	186.700	34.906	18,70	147.418	22.229	15,08	14,57
São Paulo	3.245.524	1.086.360	33,47	3.562.134	1.621.381	45,52	3.806.391	1.998.065	52,49	3.184.132	1.410.407	44,29	44,33
Tocantins	13.2791	6.231	4,69	129.782	10.620	8,18	119.059	11.972	10,06	88.405	7.202	8,15	7,66
Brasil	16.284.480	2.921.759	17,94	16.995.533	4.489.047	26,41	16.826.200	5.386.943	32,02	13.762.740	3.733.093	27,12	25,88

Fonte: beneficiárias de planos de saúde ^22^; população feminina ^23^.

* Beneficiárias de planos de saúde;

** Cobertura de plano de saúde médico.

A Tabela 4 apresenta a comparação da estimativa de abortos na saúde suplementar, utilizando o fator de correção da cobertura de planos de saúde por UF com os dados obtidos das bases de internação disponibilizada pela ANS. Para a estimativa de abortos utilizando o fator de correção, multiplicamos o número de internações por aborto (CID-10 O03 a O08) no SIH/SUS pela cobertura de planos de saúde, por faixa etária, em cada UF.

**Tabela 4:** Número de internações por aborto, segundo faixa etária e Unidade Federativa (UF), utilizando registros na base da Agência Nacional de Saúde Suplementar (ANS) ou estimativas por fator de correção. Brasil, 2015.

UF	Internações por aborto (CID-10 e/ou procedimento) registradas na saúde suplementar					Estimativa de internações por aborto na saúde suplementar usando fator de correção *					Razão de abortos estimados/abortos registrados				
	10-19 anos	20-29 anos	30-39 anos	40-49 anos	Total	10-19 anos	20-29 anos	30-39 anos	40-49 anos	Total	10-19 anos	20-29 anos	30-39 anos	40-49 anos	Total
Acre	0	10	9	4	23	8	27	22	5	63	-	2,67	2,48	1,33	2,72
Alagoas	3	56	79	13	151	60	191	129	25	404	19,85	3,41	1,63	1,88	2,68
Amazonas	6	61	82	18	167	62	192	149	30	433	10,37	3,15	1,82	1,66	2,59
Amapá	0	24	16	10	50	18	58	55	11	142	-	2,40	3,41	1,13	2,83
Bahia	36	361	566	129	1.092	170	630	640	106	1.546	4,71	1,75	1,13	0,82	1,42
Ceará	10	210	364	70	654	133	589	466	94	1.283	13,35	2,80	1,28	1,34	1,96
Distrito Federal	16	130	282	71	499	61	252	274	53	639	3,81	1,94	0,97	0,75	1,28
Espírito Santo	11	183	327	38	559	73	348	277	51	749	6,67	1,90	0,85	1,36	1,34
Goiás	19	224	290	52	585	66	295	206	29	596	3,47	1,32	0,71	0,56	1,02
Maranhão	12	94	156	41	303	48	193	119	16	377	4,02	2,06	0,76	0,39	1,24
Minas Gerais	68	712	1.291	227	2.298	407	1.627	1.496	292	3.822	5,99	2,29	1,16	1,29	1,66
Mato Grosso do Sul	8	91	121	33	253	58	154	138	23	374	7,27	1,69	1,14	0,70	1,48
Mato Grosso	4	55	80	14	153	38	152	98	16	305	9,61	2,76	1,23	1,16	1,99
Pará	25	160	206	31	422	110	343	198	34	685	4,40	2,14	0,96	1,10	1,62
Paraíba	3	66	102	23	194	47	179	156	27	409	15,63	2,72	1,53	1,19	2,11
Pernambuco	19	155	289	48	511	173	650	498	87	1.408	9,10	4,19	1,72	1,81	2,75
Piauí	8	59	91	13	171	29	109	75	12	224	3,57	1,84	0,82	0,95	1,31
Paraná	45	396	644	100	1.185	152	553	478	103	1.287	3,38	1,40	0,74	1,03	1,09
Rio de Janeiro	67	621	1.031	221	1.940	563	1.764	1.471	256	4.055	8,41	2,84	1,43	1,16	2,09
Rio Grande do Norte	10	113	221	49	393	63	228	194	37	523	6,32	2,02	0,88	0,76	1,33
Rondônia	6	74	110	20	210	19	88	55	11	173	3,09	1,19	0,50	0,55	0,82
Roraima	1	7	11	1	20	7	27	23	4	62	7,28	3,88	2,08	4,20	3,08
Rio Grande do Sul	12	161	305	52	530	120	511	546	119	1.296	10,02	3,17	1,79	2,28	2,45
Santa Catarina	14	261	506	82	863	73	325	318	55	770	5,21	1,24	0,63	0,67	0,89
Sergipe	1	33	66	11	111	51	185	160	25	422	51,40	5,60	2,43	2,32	3,80
São Paulo	263	2.368	3.920	835	7.386	1.206	4.607	4.041	891	10.745	4,58	1,95	1,03	1,07	1,45
Tocantins	2	11	16	4	33	16	64	46	8	135	8,16	5,85	2,89	1,93	4,08
Brasil	669	6.696	11.181	2.210	20.756	4.290	15.713	13.228	2.532	35.763	6,41	2,35	1,18	1,15	1,72

CID-10: Classificação Internacional de Doenças, 10ª revisão.

* Estimativa obtida pela multiplicação, por UF e faixa etária, do número de internações por aborto registradas no Sistema de Informação Hospitalar do Sistema Único de Saúde (SIH/SUS) (Tabela 1) pela cobertura de planos de saúde médicos (Tabela 3).

Verificou-se que o uso do fator de correção superestimou o número de internações por aborto na saúde suplementar no âmbito nacional, com número estimado 1,72 vezes maior do que o registrado. Foram observadas estimativas mais elevadas principalmente nas faixas etárias mais jovens, onde o número de internações estimada foi 3 a 51 vezes maior em menores de 20 anos do que o registrado. Várias UF apresentaram valor superior à média nacional, com Tocantins (4,08), Sergipe (3,80) e Roraima (3,08) apresentando os maiores valores, enquanto duas UF apresentaram estimativa de internações inferior ao registrado (Rondônia = 0,82 e Santa Catarina = 0,89). Considerando os valores obtidos, optamos por utilizar o número registrado de internações por aborto na saúde suplementar, identificados por meio da CID-10 de internação e/ou de procedimento relacionado ao aborto.

#### Etapa 3 - estimativa do fator de correção para descontar internações por aborto espontâneo

Para a exclusão de internações por complicações de aborto espontâneo, foram utilizados fatores de correção diferenciados segundo faixa etária da mulher, em virtude da maior probabilidade de ocorrência de abortos espontâneos em mulheres com idade superior a 30 anos observada em estudos prospectivos ^24^ ^,^ ^25^: fator de correção 0,90 para < 30 anos; 0,85 para mulheres de 30-39 anos; e 0,75 para mulheres com 40 anos ou mais.

Esses fatores de correção foram aplicados separadamente às internações por aborto no setor público e privado, já que a distribuição por faixa etária se mostrou diferente nos dois setores, com maior número de internações nas faixas etárias mais jovens no setor público, e nas faixas maiores de 30 anos no setor privado (Tabelas 1 e 2).

#### Etapa 4 - estimativa do fator de correção para complicações que não resultaram em internação hospitalar

Para correção de abortos que não resultaram em internação hospitalar, a metodologia proposta pelo Instituto Guttmacher recomenda utilizar a opinião de especialistas para estimar o tipo de prestador e método utilizado, a taxa de complicação com cada método e a proporção de internações no caso de complicações para quatro subpopulações: pobre urbana, não pobre urbana, pobre rural e não pobre rural ^16^.

Optamos por utilizar os dados da PNA 2016 para orientar a escolha do fator de correção, com posterior consulta a especialistas para discutir os valores obtidos. Na PNA, foi utilizado o método direto para estimativa do número de abortos, por meio de entrevista com as mulheres, e uso do método de urna, estratégia de coleta que visa reduzir a subnotificação do aborto induzido, já que é feita de forma não identificada, preservando o anonimato da mulher ^4^. A estimativa da PNA 2016 do número de mulheres que realizou aborto em 2015 foi utilizada, portanto, como valor de referência para a definição do fator de correção, a partir de sua comparação com o número de internações por aborto no setor público e privado.

Foram simulados alguns cenários, visando obter fatores de correção que contemplassem variações nos parâmetros utilizados. A comparação dos cenários está descrita na Tabela 5. Em todos os cenários, consideramos apenas as internações de mulheres de 18-39 anos, faixa etária adotada na PNA, e excluímos as prováveis internações por aborto espontâneo, após aplicar o fator de correção específico para cada faixa etária apresentado na etapa 3. A faixa etária de 18 e 19 anos não está disponível na base da ANS, apenas a faixa de 15-19 anos, sendo necessário estimar a proporção de internações na faixa de 15-19 anos que ocorre em jovens de 18 e 19 anos. Segundo a PNA, a população feminina de 18-39 anos, em 2015, era de 37.287.746 mulheres. Subtraindo desse total as mulheres com 20-39 anos (34.032.349), tem-se que as 3.255.397 mulheres restantes correspondem a 38% da população estimada na faixa etária de 15-19 anos (8.538.652). Como a frequência de aborto no último ano aumenta com o incremento da idade na faixa etária de 15-19 anos (segundo dados da PNA, 7,6% em jovens de 12-15 anos, 10,4% em jovens de 16-17 anos e 11,2% em jovens de 18 e 19 anos) ^4^, consideramos que a proporção de internações em mulheres de 18 e 19 anos seria maior do que a distribuição populacional, sendo utilizado o parâmetro de 75% das internações na faixa etária de 15-19 anos como estimativa do número de internações em mulheres com 18 e 19 anos.

**Tabela 5:** Fator de correção para complicações de aborto que não resultaram em internação hospitalar no Sistema Único de Saúde (SUS) e na saúde suplementar, em mulheres de 18-39 anos. Brasil, 2015.

	Número total mulheres que relataram aborto no último ano estimados pela PNA 2016	Estimativa do número de mulheres que relataram abortos na PNA em usuárias SUS *	Estimativa do número de mulheres que relataram abortos na PNA em usuárias da saúde suplementar *	Número de internações por aborto no SUS após aplicação do fator de correção aborto espontâneo **	Número de internações por aborto na saúde suplementar após aplicação do fator de correção aborto espontâneo **	Número total de internações por aborto após aplicação do fator de correção aborto espontâneo**	Fator de correção SUS	Fator de correção saúde suplementar	Fator de correção total
	(a)	(b)	(c)	(d)	(e)	(f)	(b/d)	(c/e)	(a/f)
Cenário 1	503.000	-	-	103.623	15.963	119.586	-	-	4,2
Cenário 2		371.208	128.803	103.623	15.963	-	3,6	8,1	-
Cenário 3a		428.635	74.365	103.623	15.963	-	4,1	4,7	-
Cenário 3b		399.131	103.869	103.623	15.963	-	3,9	6,5	-
Cenário 3c		383.301	119.699	103.623	15.963	-	3,7	7,5	-

PNA: *Pesquisa Nacional de Aborto*.

* Para estimar o número de abortos em usuárias do SUS e da saúde suplementar, a partir do número estimado de mulheres que realizaram aborto no último ano obtido pela PNA, foram considerados os seguintes cenários: cenário 1: considerado o total de mulheres que realizaram abortos pela PNA em 2015 e a soma do total de internações por aborto no setor público e privado no mesmo ano, após aplicação do fator de correção para descontar internações por aborto espontâneo; cenário 2: utilizada a cobertura de planos de saúde por faixa etária (18% em mulheres de 18 e 19 anos; 26% em mulheres de 20-29 anos, 32% em mulheres de 30-39 anos) para estimar o número de usuárias da saúde suplementar e do SUS e considerada a mesma frequência de mulheres que relataram aborto no último ano estimada pela PNA 2016 (1,35%) em ambos os setores (SUS e saúde suplementar); cenário 3: utilizada a cobertura de planos de saúde por faixa etária (18% em mulheres de 18 e 19 anos; 26% em mulheres de 20-29 anos, 32% em mulheres de 30-39 anos) para estimar o número de usuárias da saúde suplementar e do SUS e consideradas frequências diferentes de mulheres que relataram aborto no último ano em usuárias do SUS e da saúde suplementar (cenário 3a: frequência de mulheres que relataram aborto no último ano na saúde suplementar = 50% da frequência em usuárias dos SUS [1,56% SUS; 0,78% saúde suplementar]; cenário 3b: frequência de mulheres que relataram aborto no último ano na saúde suplementar = 75% da frequência em usuárias dos SUS [1,45% SUS; 1,09% saúde suplementar]; cenário 3c: frequência de mulheres que relataram aborto no último ano na saúde suplementar = 90% da frequência em usuárias dos SUS [1,39% SUS; 1,25% saúde suplementar]);

** Para estimar internações em mulheres de 18 e 19 anos, foi considerado que 75% das internações em mulheres de 15-19 anos ocorreram em mulheres entre 18 e 19 anos. Fator de correção utilizado para descontar internações por aborto espontâneo: 18-29 anos = 0,90; 30-39 anos = 0,85 ^24^ ^,^ ^25^.

No cenário 1, estimamos o fator de correção analisando todas as mulheres conjuntamente, ou seja, sem fazer distinção entre usuárias do SUS e da saúde suplementar e considerando que o perfil de realização de aborto é semelhante entre esses dois grupos. O total estimado, pela PNA, de mulheres que fizeram aborto em 2015 (503 mil) foi comparado ao total de internações públicas e privadas por aborto não espontâneo em mulheres de 18-39 anos.

No cenário 2, utilizamos a cobertura de planos de saúde, por faixa etária, na população de 18-39 anos, para estimar o número de abortos em mulheres usuárias do SUS e da saúde suplementar, considerando o mesmo perfil de realização de abortos nos dois grupos. A PNA estimou uma taxa de realização de aborto no último ano de 1,35%, e utilizamos esse valor para estimar o número de abortos em usuárias do SUS e do saúde suplementar, com posterior comparação com o número de internações por aborto em cada setor, calculando um fator de correção específico para usuárias do SUS e da saúde suplementar.

Por fim, no cenário 3, consideramos que a frequência de realização de aborto é diferente em usuárias do setor público e privado, já que a PNA mostrou que mulheres de maior renda e escolaridade têm menor frequência de abortos, perfil observado também em beneficiárias de planos de saúde,. Utilizamos três parâmetros distintos: frequência 50% menor de aborto em usuárias da saúde suplementar (diferença observada nos grupos de maior e menor renda da PNA) e frequência 25% e 10% menor, considerando que as usuárias do setor público e privado não estão apenas nesses extremos (Tabela 5: cenários 3a, 3b e 3c, respectivamente). Com esses parâmetros, estimamos o número de mulheres usuárias do SUS e da saúde suplementar, entre as mulheres que relataram aborto no último ano estimadas pela PNA, e comparamos com as internações registradas no setor público e privado, gerando fator de correção específico para cada setor.

Os diferentes cenários mostram fator de correção próximo a 4 para usuárias do SUS. Já em usuárias do saúde suplementar, o fator de correção varia de 4,2 (utilização de fator único para o total de internações SUS e saúde suplementar) a 8,1, cenário, em que se considera similar a frequência de aborto em usuárias do SUS e da saúde suplementar.

#### Etapa 5 - estimativa do número de abortos induzidos

A fórmula para cálculo do número de abortos induzidos é:

Número de abortos induzidos = (número de internações com financiamento público × fator de correção para abortos espontâneos × fator de correção para complicações sem internação) + (número de internações na saúde suplementar × fator de correção para abortos espontâneos × fator de correção para complicações sem internação) ^16^.

O fator de correção utilizado para descontar abortos espontâneos variou de acordo com a faixa etária da mulher, conforme informado na etapa 3. O fator de correção para complicações que não resultaram em internação, a ser utilizado para todas as faixas etárias, foi de 4 para usuárias do SUS e de 5 para usuárias da saúde suplementar (Tabela 6). A escolha do fator de correção para usuárias da saúde suplementar considerou o cenário mais conservador, em que a frequência de abortos em usuárias do saúde suplementar é 50% menor do que em usuárias do SUS.

**Tabela 6:** Cálculo da estimativa do número de abortos induzidos, da taxa de abortos induzidos por 1.000 mulheres de 10-49 anos e da razão de abortos induzidos por 100 nascidos vivos segundo sistema de atenção à saúde. Brasil, 2015.

Sistema de atenção à saúde/Idade da mulher (em anos)	Número de internações por aborto *	Número internações após aplicação do fator de correção para abortos espontâneo **	Fator de correção para complicações (LI/LS) ***	Total estimado de abortos induzidos (LI/LS) ^#^	População feminina de 10-49 anos ^##^	Nascidos vivos em mulheres de 10-49 anos ^##^	Taxa de abortos induzidos/1.000 mulheres 10-49 anos (LI/LS) ^#^	Razão de abortos/100 nascidos vivos (LI/ LS) ^#^
	(a)	(b)	(c)	(d) = b × c	(e)	(f)	(d/e) × 1.000	(d/f) × 100
SUS								
10-29	83.400	75.060						
30-39	41.319	35.121						
40-49	9.335	7.001						
Total	134.054	117.182	4 (3/5)	468.728 (351.546/585.910)	47.418.584	2.221.005	9,9 (7,4/12,4)	21,1 (15,8/26,4)
Saúde suplementar								
10-29	7.365	6.629						
30-39	11.181	9.504						
40-49	2.210	1.658						
Total	20.756	17.791	5 (4/6)	88.955 (71.164/106.746)	16.450.369	796.198	5,4 (4,3/6,5)	11,2 (8,9/13,4)
Brasil				557.683 (422.710/692.656)	63.868.953	3.017.203	8,7 (6,6/10,8)	18,5 (14,0/23,0)

LI: limite inferior; LS: limite superior; SUS: Sistema Único de Saúde.

* No SUS: número de internações por aborto corresponde às internações no Sistema de Informação Hospitalar (SIH/SUS) com CID-10 O03 a O08; na saúde suplementar: número de internações por aborto corresponde às internações na base da Agência Nacional de Saúde Suplementar (ANS) com CID-10 O03 a O08 ou procedimentos “31309020” - aspiração manual intrauterina (AMIU) pós-abortamento ou “31303013” - AMIU ou “31309062” - curetagem pós-abortamento;

** Fator de correção diferenciado segundo faixa etária (10-29 anos = 0,90; 30-39 anos = 0,85; 40-49 anos = 0,75), considerando as referências utilizadas ^24^ ^,^ ^25^;

*** Fator de correção apresentado na Tabela 5. Para a saúde suplementar considerando o cenário mais conservador que considera uma frequência de aborto 50% menor em mulheres da saúde suplementar em relação ao SUS;

^#^ Para cálculo dos limites inferior e superior do total estimado de abortos induzidos foram utilizados os fatores de correção (LI/LS) informados entre parênteses na coluna c. Para cálculo dos limites inferior e superior da taxa de aborto induzido e da razão de aborto inseguro, foi utilizado o total estimado de abortos inseguros obtidos com a aplicação dos fatores de correção para LI e LS;

^##^ Para estimativa da população feminina e número de nascidos vivos usuários da saúde suplementar, foi utilizada a cobertura de planos de saúde médicos, por faixa etária, em mulheres de 10-49 anos no ano de 2015 (10-19 anos = 18%; 20-29 anos = 26%; 30-39 anos = 32%; 40-49 anos = 27%) obtida no *site* da ANS ^22^. População feminina e nascidos vivos SUS foram obtidos pela subtração das beneficiárias de planos de saúde do número total da população feminina e do total de nascidos vivos em 2015. População feminina de 10-49 anos obtida em Departamento de Informática do SUS ^23^, e número de nascidos vivos obtido em Departamento de Informática do SUS ^33^.

#### Etapa 6 - cálculo da taxa de aborto induzido por 1.000 mulheres em idade fértil e da razão de abortos induzido por 100 nascidos vivos

O numerador de ambos os indicadores foi o número de abortos induzidos estimado na etapa 5. Para o denominador do primeiro indicador, foi utilizado o número estimado de mulheres com 10-49 anos, atualizado após o *Censo Demográfico* 2022, obtido no site do DATASUS ^23^. Para o segundo indicador, foi utilizado o número de nascidos vivos obtido do Sistema Nacional de Informações sobre Nascidos Vivos (SINASC), captados pelo pacote microdatasus por meio da linguagem de programação estatística R ^18^. Para cálculo da população feminina de 10-49 anos e de nascidos vivos assistidos na saúde suplementar, utilizou-se a cobertura de planos de saúde por faixa etária obtida no site da ANS. Para ambos os indicadores, foi calculado o limite superior (LS) e inferior (LI), aumentando e diminuindo em uma unidade o fator de correção para complicações que não resultaram em internação.

A taxa de aborto induzido total foi de 8,7 por 1.000 mulheres em idade fértil (LI = 6,6; LS = 10,8), sendo de 9,9 (LI = 7,4; LS = 12,4) em usuárias do SUS e de 5,4 (LI = 4,3; LS = 6,5) em usuárias da saúde suplementar. Já a razão de abortos induzido total foi de 18,5 por 100 nascidos vivos (LI = 14,0; LS = 23,0), com valores de 21,1 (LI = 15,8; LS = 26,4) em usuárias do SUS e 11,2 (LI = 8,9; LS = 13,4) em usuárias da saúde suplementar.

## Discussão

O aborto é um importante problema de saúde pública, e quantificar e monitorar sua ocorrência é uma estratégia relevante para a formulação e a implantação de ações e serviços de saúde reprodutiva e de atenção às mulheres em situação de abortamento. A vantagem do método indireto é permitir a estimativa do número de abortos induzidos a partir de dados administrativos que estão disponíveis de forma rotineira e gratuita. Entretanto, suas limitações estão relacionadas justamente à qualidade do registro nessas bases de dados e aos parâmetros utilizados para descontar internações por abortos espontâneos e para estimar as complicações de aborto que não necessitaram de internação, discutidos a seguir.

Dados de internação no setor público são utilizados desde os anos 1990. Um estudo de validação de âmbito nacional, realizado nos anos 2021-2022, identificou boa cobertura de registro de internações obstétricas no SIH/SUS, sem evidência de sub-registro de internações por aborto ao utilizar CID-10 de internação ^26^. Informações sobre internações na saúde suplementar não estavam disponíveis durante a realização dos últimos estudos de âmbito nacional que estimaram o número de abortos induzidos pelo método indireto ^5^ ^,^ ^6^. Em ambos, utilizou-se fator de correção para estimar o número de internações por aborto na saúde suplementar a partir do número de internações observado no setor público e da cobertura de planos de saúde no país.

Entretanto, o uso desse fator de correção apresenta dois problemas. Em primeiro lugar, o uso de um único fator de correção nacional desconsidera a existência de diferenças regionais no acesso aos planos de saúde. Coberturas mais elevadas são observadas nas regiões Sul, Sudeste e Centro-oeste ^27^, o que pode gerar estimativas enviesadas, com sobre-registro de abortos induzidos em estados com cobertura de plano de saúde mais baixa que a média nacional e sub-registro naqueles com cobertura acima da média. O segundo ponto é que o uso desse fator de correção parte da premissa de que a frequência de abortos induzidos é igual entre usuárias do SUS e da saúde suplementar. Dados das PNA 2010 ^3^ e 2016 ^4^ mostram que a frequência de abortos é muito menor em mulheres de maior escolaridade e renda, perfil encontrado em usuárias do setor suplementar, o que nos faz supor que a frequência de aborto seria menor entre essas usuárias, por maior acesso a serviços de planejamento reprodutivo e menor ocorrência de gestações não intencionais.

Neste estudo, utilizamos, pela primeira vez, os dados de internação hospitalar em serviços privados disponibilizados pela ANS a partir de 2015. Devido ao sub-registro da CID-10 nessa base, avaliamos também dados de procedimentos relacionados ao aborto, o que resultou em aumento de 100% das internações na saúde suplementar. Esses registros incluem tanto procedimentos realizados durante internação hospitalar quanto na modalidade hospital-dia. Ressaltamos que o uso de procedimentos de aborto não é recomendado pela metodologia do Instituto Guttmacher ^16^, mas foi utilizado para corrigir o sub-registro identificado. Entretanto, é possível que exista algum sobre-registro, caso mulheres admitidas por diagnósticos relacionados às CID-10 O00 a O02 tenham sido submetidas a procedimento de aborto e não tenham CID-10 registrada, sendo incluídas de forma indevida no total de internações por aborto.

A comparação do número de registros nessas bases com o número estimado de internações na saúde suplementar, calculado a partir do número de internações no setor público e da cobertura de planos de saúde, mostrou que o cálculo com uso do fator de correção superestimou as internações no setor privado em todas as UF, exceto em Santa Catarina e Rondônia. Ressalta-se que, diferentemente de estudos anteriores ^5^ ^,^ ^6^, foram utilizados fatores de correção específicos por UF e faixa etária, o que permitiu o cálculo de estimativas mais precisas, considerando o contexto de cada local. 

Também diferentemente dos estudos anteriores de âmbito nacional ^5^ ^,^ ^6^, utilizamos fator de correção específico por faixa etária para descontar abortos espontâneos. Fatores diferenciados por faixa etária já haviam sido utilizados em investigação no Rio de Janeiro ^20^, mas com valores diferentes dos adotados neste estudo. Como a base da ANS só fornece a faixa etária de 30-39 anos, não foi possível utilizar fatores específicos para mulheres com idade acima de 35 anos, na qual frequência de abortos espontâneos se eleva. É provável, assim, que a estimativa de abortos espontâneos na faixa etária de 30-34 anos esteja superestimada, devido ao uso do mesmo fator de correção utilizado para mulheres de 35-39 anos.

Por fim, o parâmetro mais complexo a ser utilizado é o fator de correção para complicações que não resultam em internação. Resultados das PNA 2010 ^3^ e 2016 ^4^ indicam que cerca de metade das mulheres brasileiras que tiveram aborto induzido precisaram ser hospitalizadas, sendo sugerido fator de correção de 2. Entretanto, a PNA incluiu apenas mulheres de 18-39 anos e não avaliou aquelas residentes em região rural e analfabetas. Portanto, das quatro subpopulações sugeridas pela metodologia Guttmacher ^16^, apenas a subpopulação não pobre urbana e pobre urbana estariam representadas, sendo esta última incompleta pela não inclusão das analfabetas. O número estimado de 503 mil mulheres que realizaram aborto na PNA 2016 ^4^ é uma extrapolação de que as mulheres não incluídas tenham a mesma frequência de aborto e o mesmo padrão de utilização de serviços de saúde das entrevistadas.

No atual estudo, foram feitas algumas simulações e em nenhuma delas foi obtido o fator de correção 2, sugerido pela PNA 2016 ^4^. Em todos os cenários, o fator de correção para o setor público foi próximo a 4, semelhante ao utilizado nos estudos nacionais anteriores ^5^ ^,^ ^6^. Já para a saúde suplementar, o fator de correção variou de 4,2 a 8,1, dependendo dos pressupostos de população coberta por planos de saúde e frequência de aborto em usuárias do SUS e da saúde suplementar. Vale ressaltar que, para essa análise de cenários, consideramos apenas as internações de mulheres de 18-39 anos, faixa etária estudada nas PNA. Para isso, estimamos que 75% das internações na faixa etária de 15-19 anos ocorreu em jovens de 18 e 19 anos. Caso seja adotada proporção menor, o fator de correção seria ainda superior, pois o número de internações em mulheres de 18 e 19 anos seria menor.

A escolha dos fatores de correção, principalmente do utilizado para as complicações que não resultaram em internação, é motivo de muito debate, pois depende da segurança dos métodos utilizados para interrupção da gestação e do acesso aos serviços de saúde ^28^ ^,^ ^29^. Quanto mais inseguros os métodos, maior a proporção de mulheres que necessitarão de internação ^30^ e menor será esse fator de correção. As PNA mostraram declínio na proporção de mulheres que usaram medicamento para a interrupção da gestação (48% em 2010; 39% em 2021), método seguro e com baixa taxa de complicação, e também da proporção de mulheres que foram hospitalizadas para finalizar o aborto (55% em 2010; 43% em 2021) ^3^ ^,^ ^31^.

Devido às incertezas dos parâmetros utilizados, a metodologia do Instituto Guttmacher recomenda o cálculo dos limites superior e inferior, aumentando e diminuindo em uma unidade o fator de correção para complicações que não resultaram em internação. Esse cálculo visa produzir estimativas baixa e alta e se espera que a incidência real de aborto esteja dentro dessa faixa ^16^. Para as internações no setor público, nossa proposta é que seja utilizado fator de correção 4, com variação de 3 a 5; enquanto, para o setor privado, fator de correção 5, com variação de 4 a 6.

Com os cálculos empregados, estimamos taxa de aborto induzido de 8,7 por 1.000 mulheres em idade fértil em 2015, valor muito inferior ao coeficiente médio anual observado entre 1996-2012, de 17 por 1.000 ^5^. Entretanto, nosso limite superior (10,8) foi próximo ao limite inferior registrado em 2013, de 12 por 1.000 ^6^. Já para a razão de abortos induzidos, nossa estimativa de 18,5 por 100 nascidos vivos foi metade do valor médio estimado para o período 1996-2012. A tendência de queda da taxa de aborto induzido identificada entre 1995 e 2013 ^5^ ^,^ ^6^ seria uma possível explicação para o menor valor observado da taxa de aborto induzido neste estudo, mas não para a razão de aborto induzido, que apresentou estabilidade no período de 1996-2012. Diferenças nos métodos de cálculo adotados neste estudo, como o uso de dados de internação da ANS, fatores específicos por faixa etária para descontar abortos espontâneos e fatores de correção diferenciados para internações no setor público e na saúde suplementar são os motivos mais plausíveis para as discrepâncias encontradas.

A grande diferença do fator de correção utilizado para complicações que não resultaram em internação em relação ao fator proposto pela PNA 2016 ^4^ tem algumas possíveis explicações. Em primeiro lugar, existe a possibilidade de que o número de internações esteja subestimado devido às falhas de registro ou à glosa das autorizações de internação. É possível também que, em internações de mulheres com complicações tardias do aborto, não tenha sido registrada a CID-10 de aborto, mas a CID-10 da complicação que motivou a internação. Óbitos por aborto não são incluídos nessa metodologia, o que pode resultar em sub-registro, caso não conste CID-10 de abortamento na internação que resultou no óbito. O número de abortos espontâneos na faixa etária de 30-34 anos pode estar superestimado, reduzindo o número estimado de internações por aborto nessa faixa etária, tanto no SUS quanto na saúde suplementar. Em relação às estimativas de aborto, a PNA extrapolou seus resultados para toda a população feminina de 18-39 anos, valor que comparamos ao número total de registros de internação hospitalar. É possível que a frequência de abortos e o perfil de utilização de serviços seja diferente em mulheres analfabetas e residentes em região rural, resultando num fator de correção maior, principalmente em usuárias do SUS. Existe, ainda, a possibilidade de que as mulheres tenham respondido à pergunta sobre hospitalização na PNA de forma imprecisa, sendo captados procura e atendimento em serviço de saúde, e não necessariamente internação hospitalar. Usuárias da saúde suplementar podem ter mais acesso a métodos mais seguros, fazendo com que tenham menos necessidade de cuidado hospitalar pós-abortamento. A PNA estimou o número de mulheres que realizou aborto e não o número de abortos, podendo haver mais de um aborto de uma mesma mulher no mesmo ano, estando o número de abortos subestimado. Por fim, internações em serviços privados com pagamento por desembolso direto não estão incluídos na base da saúde suplementar, que só contempla internações pagas por planos de saúde.

Uma questão que merece atenção em estudos futuros é a distribuição dos abortos por faixa etária. Nas simulações realizadas, foram consideradas apenas as internações em mulheres de 18-39 anos, faixa etária utilizada na PNA. Entretanto, dados das PNA 2010, 2016 e 2021 mostram que, embora a maior ocorrência de abortos seja na faixa de 20-29 anos, é na faixa de 15-19 anos que a realização do primeiro aborto é mais frequente ^3^ ^,^ ^4^ ^,^ ^31^. Dados nacionais mostram frequência maior de abortos induzidos em jovens de maior escolaridade, em decorrência de a gravidez precoce competir com outros projetos pessoais ^32^, sendo possível que a frequência de aborto em adolescentes seja diferente entre usuárias do SUS e da saúde suplementar.

Por fim, num país tão grande e diverso como o Brasil, é fundamental que estudos de âmbito local e regional forneçam evidências para embasar a utilização de fatores de correção mais adequados para cada contexto. Dados mais específicos por regiões do país e por tipo de prestador são necessários para fundamentar a definição do fator de correção. Diferentes valores resultarão em estimativas diferentes do número de abortos induzidos, limitando a comparação dos resultados.

## Conclusão

A metodologia de cálculo do número de abortos induzidos indiretamente, a partir das internações hospitalares por complicações do aborto, permite obter estimativas rápidas com bases de dados administrativos de acesso rotineiro e gratuito. Neste estudo, propusemos a revisão da metodologia utilizada previamente, incorporando a base de dados de internações hospitalares do setor suplementar e de fatores de correção para abortos espontâneos e para complicações que não resultaram em internação no setor público e privado. Essa nova abordagem permite estimar o número de abortos induzidos para toda a população feminina, bem como para as usuárias do SUS e da saúde suplementar separadamente. Entendemos que essa metodologia pode ser aprimorada com o refinamento dos fatores de correção e com a melhoria contínua das bases de dados utilizadas. Investigações futuras devem priorizar o estudo do aborto em adolescentes e obtenção de parâmetros para o cálculo dos fatores de correção para populações específicas.
